# Structural basis for inhibition of *Mycobacterium tuberculosis* α-methylacyl-CoA racemase by 2-arylthiopropanoyl-CoA inhibitor analogs

**DOI:** 10.1016/j.jbc.2025.110848

**Published:** 2025-10-22

**Authors:** Otsile O. Mojanaga, Timothy J. Woodman, Matthew D. Lloyd, K. Ravi Acharya

**Affiliations:** Department of Life Sciences, University of Bath, Bath, United Kingdom

**Keywords:** α-Methylacyl-CoA racemase (AMACR, P504S), 2-arylthiopropanoyl-CoA inhibitor, CoA-transferase, ibuprofen, kinetic study, *M. tuberculosis*, X-ray crystallography

## Abstract

α-Methylacyl-CoA racemase (AMACR; P504S) is a pivotal enzyme involved in the β-oxidation of branched-chain fatty acids and bile acid intermediates, catalyzing the conversion between (2*R*)- and (2*S*)-2-methylacyl-CoA thioester epimers. The AMACR reaction enables downstream catabolism of these thioesters *via* stereospecific enzymes within the β-oxidation pathway. The AMACR homolog in *Mycobacterium tuberculosis* (MCR) has emerged as a tractable model for dissecting the mechanistic underpinnings of the racemization reaction and presents a promising therapeutic target given the pathogen’s dependence on lipid metabolism for persistence and virulence. Previously, we reported the detailed molecular structure of wild-type MCR and in complex with a diverse set of acyl-CoA substrates. They revealed conserved active site residues that mediate substrate anchoring and epimerization and highlighted distinct molecular interactions that confer selectivity toward 2-methyl-branched substrates. Complementing these results, in this report, we present high-resolution structures for 2-arylthiopropanoyl-CoA inhibitors in complex with MCR and a comprehensive set of enzyme inhibition assays to delineate structure-activity relationships and probe competitive binding modes. Our findings underscore the importance of inhibitor side-chain branching and CoA anchoring in modulating enzymatic turnover and inhibition. Together, these data enhance our understanding of the racemization mechanism of MCR and establish a structural foundation for the rational design of selective inhibitors. Targeting MCR could represent a novel future therapeutic strategy for *M. tuberculosis* based on impairing cholesterol utilization.

Branched-chain fatty acids are prevalent in the human diet and are synthesized endogenously *via* cholesterol metabolism. Their degradation follows a stereoselective β-oxidation pathway, which preferentially metabolizes *S*-2-methylacyl-CoA intermediates, which requires *R*-2-methylacyl-CoA esters (from dietary sources or bile acid biosynthesis) to undergo stereochemical conversion into the *S*-epimer. This epimerization is catalyzed by α-methylacyl-CoA racemase (AMACR; P504S), an enzyme that facilitates the interconversion of 2-methyl-branched acyl-CoAs, allowing their entry into β-oxidation (1, 2, 3). Beyond its metabolic role, AMACR contributes to the bioactivation of nonsteroidal anti-inflammatory drugs (NSAIDs), such as ibuprofen (1, 2, 3).

In *Mycobacterium tuberculosis* (*Mtb*), the causative agent of tuberculosis, cholesterol metabolism is crucial for intracellular survival and virulence (4, 5). Cholesterol is one of the several carbon sources which can be utilized during initial *Mtb* infection, but it is essential for growth and persistence during chronic infection (5, 6). Cholesterol is converted into cholest-4-en-3-one before the C26 side-chain methyl group is hydroxylated by cytochrome P_450_ enzymes CYP125A1 (7) or CYP142A1 (8), giving the 25*S*- and 25*R*-hydroxylated products, respectively. The resulting alcohols are further oxidized to the corresponding aldehydes and carboxylic acids (7, 8) in this Ω-oxidation pathway. These carboxylic acids are subsequently converted into acyl-CoA thioesters by the non-stereoselective acyl-CoA synthetase fadD19 (Fig. 1*A*) (4). Sulfate and propanoate esters of cholesterol are also an energy source for *Mtb*. These esters are exclusively hydroxylated by CYP142A1, as CYP125A1 is unable to convert them (9), with subsequent conversion to the corresponding *R*-2-methylacyl-CoA esters (Fig. 1*B*). The stereochemistry of these intermediates is critical as the generated 25*R* α-methylacyl-CoAs must be epimerized into the 25*S*- equivalents to enter β-oxidation (4, 10).

**Figure 1:**
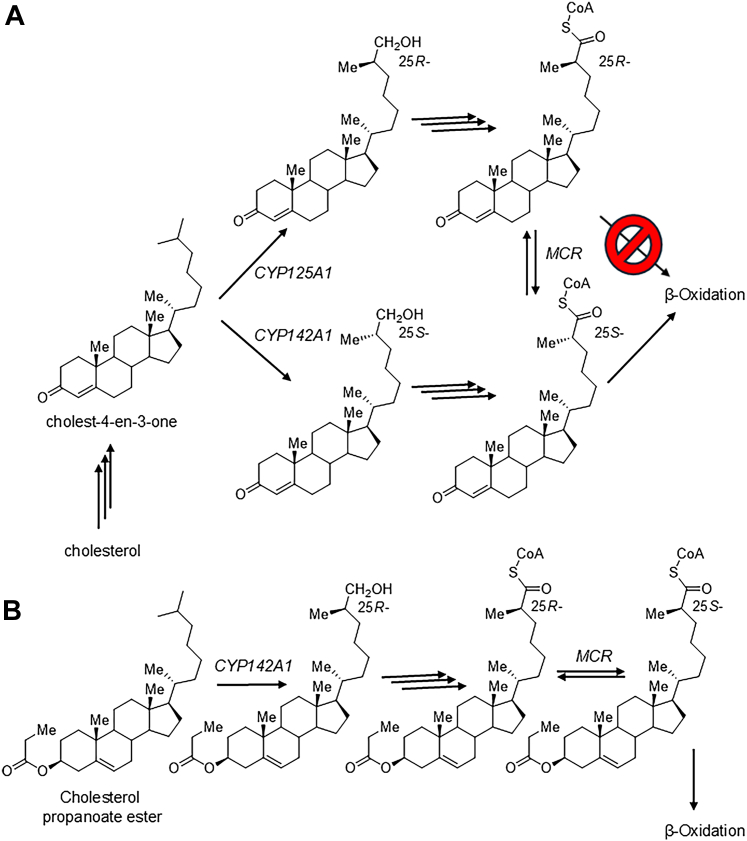
**Cholesterol metabolism by *Mycobacterium tuberculosis*.** Cholesterol is converted into an epimeric mixture of bile acids *via* hydroxylation by cytochrome P_450_ enzymes, followed by acyl-CoA formation. *A*, metabolism of cholesterol; *B*, metabolism of cholesterol propanoate ester. The first step of β-oxidation is performed by an acyl-CoA oxidase which is stereoselective for the 25*S*- substrate epimer (10). Degradation of the 25*R*-epimer requires conversion to the 25*S*-epimer by MCR in both pathways (4). CYP, cytochrome P_450_ enzyme; MCR, α-methylacyl-CoA racemase from *M. tuberculosis*.

*Mtb* encodes two putative α-methylacyl-CoA racemases (*mcr* and *far*), with α-methylacyl-CoA racemase (MCR) functionally linked to bile acid-derived acyl-CoA epimerization (4, 11). The racemase activity of MCR has been confirmed through HPLC (4), isotope-exchange (12, 13, 14), and colorimetric assays (12). *In vitro* assays have demonstrated that MCR can epimerize α-methylacyl-CoAs derived from 25*R*-bile acids (4). Although fatty acid racemase (FAR) adopts a typical family III CoA transferase fold (15) and has the required active site bases (Supplementary Information, Fig. S1), its enzymatic function still requires experimental confirmation. In addition, *M. tuberculosis* also encodes for *Rv3272*, a sequence-related putative family III CoA transferase (Supplementary Information, Fig. S1) (16). This latter enzyme lacks the required catalytic bases for α-methylacyl-CoA racemase activity.

Structurally, MCR functions as a homodimer (∼84–89 kDa) (12, 13, 14, 17), classified within the family III CoA transferases. Its active site, located at the dimer interface, consists of key catalytic residues contributed by both monomers. Previous crystallographic studies have implicated a His126/Glu241 catalytic dyad (or alternatively Asp156) in proton abstraction and reprotonation during racemization (12, 13, 14). This mechanism was originally proposed to involve substrate repositioning across a hydrophobic methionine-rich surface (13), although the precise molecular determinants governing substrate recognition and inhibition remain unclear.

We previously reported the crystal structure of wild-type MCR along with selected active site mutants in a newly identified crystal form (12). We subsequently reported a second novel crystal form of MCR, providing structural insights into its function (17). We presented the apo-enzyme structure of this second new crystal form, alongside 12 cocrystal structures of MCR bound to a series of acyl-CoA esters, including ibuprofenoyl-CoA and analogs, and various acyl-CoAs with linear and branched alkyl side-chains. These structural datasets significantly improve our understanding of active site interactions, revealing key mechanistic details of MCR-mediated racemization and ligand binding. These structures reveal four acyl-CoA substrate binding subsites *viz.* the CoA binding pocket, the C_α_-methyl binding pocket, the catalytic center, and the side-chain binding pocket (17).

These structures are entire consistent with a 1,1-proton transfer mechanism (13) in which the substrate α-proton is removed by the active site bases [Asp156 or the His126/Glu241 dyad (12, 13, 17)] to form an enolate intermediate (18). The *p*K_a_ for the 2-methylacyl-CoA α-proton is *ca*. 21 (19), which enables the formation of a discrete (enolate) intermediate rather than requiring a concerted mechanism (3). The stereochemical outcome of the reprotonation step of the MCR-catalyzed reaction has not been determined, but mechanistic studies on human AMACR [which has 43% sequence identity (14)] have shown that a near 1-to-1 mixture of product epimers is obtained (20, 21). Notably, our findings challenge the previously proposed “mirror image packing” model (13), which suggested extensive substrate repositioning for proper binding. Instead, our structural data supported an alternative “enantiomer superposition” model, wherein both *R*- and *S*- epimers can be accommodated through subtle conformational rearrangements, rather than requiring extensive repositioning of the substrate’s side-chain (17). This revised model suggests greater catalytic flexibility in MCR than previously recognized, allowing efficient interconversion between stereoisomers without major structural shifts. This mechanistic model is also consistent with observations for other cofactor-independent racemases and epimerases (22).

Beyond structural insights, we also reported inhibition data for those acyl-CoAs, assessing their potency and interactions within the active site. Our structure-activity relationship analysis links substrate physicochemical properties to binding potency, providing a mechanistic framework for understanding inhibitor development. Prior studies highlighted lipophilic side-chains as critical determinants of substrate binding and inhibition, observations that remain consistent for both AMACR and MCR (1, 2, 3, 23, 24). Our results refine this perspective, elucidating how side-chain variations modulate enzyme-substrate interactions.

Given MCR’s key role in cholesterol metabolism (4), inhibiting this enzyme presents a potential therapeutic target during the chronic infection stage where cholesterol is required for persistence (5, 6). 25*R-*Bile acids are exclusively produced by CYP142A1 (8) (Fig. 1) and MCR activity is essential for their β-oxidation. Inhibition of MCR is expected to block 25*R*-bile acid metabolism. Although *Mtb* growth inhibition by blocking MCR activity has not yet been demonstrated, genetic knock-down of CYP125A1, an upstream enzyme in cholesterol metabolism (Fig. 1*A*), did show the desired effect (7). While inhibition of human AMACR has been extensively studied (1, 2, 3, 23, 24, 25, 26, 27, 28, 29, 30, 31) due to its overexpression in prostate and other cancers, equivalent research on *Mtb* MCR remains scarce (3, 32, 33). The absence of systematic structure-based inhibitor analysis has hindered efforts to design selective compounds tailored to *Mtb*-specific metabolic pathways.

In our new study, we address this gap by providing high-resolution structural data of thirteen 2-arylthiopropanoyl-CoA inhibitors (Fig. 2) (23) in complex with MCR, alongside quantitative binding and inhibition data. The presence of the sulfur atom adjacent to the α-carbon is thought to result in diminution of the α-proton *p*K_a_ value by ∼5 *p*H units (23) [by analogy to the change in α-proton *p*K_a_ value from ∼21 for octanoyl-CoA (19, 34) to ∼16 for 3-thiaoctanoyl-CoA (34)], enhancing formation of the enolate intermediate and inhibitor binding. Facile exchange of 2-arylthiopropanoyl-CoA α-protons in the absence of AMACR can be readily observed (23). Inhibition of human AMACR 1A by 2-arylthiopropanoyl-CoAs has been previously reported (23), but a structural understanding of inhibitor binding is lacking. The findings in this study enhance our understanding of *Mtb* cholesterol metabolism, refining the molecular basis for MCR substrate specificity and enzyme function. Furthermore, by identifying key determinants of substrate and inhibitor affinity and conformational adaptability, we lay the foundation for future drug discovery efforts targeting MCR.

**Figure 2:**
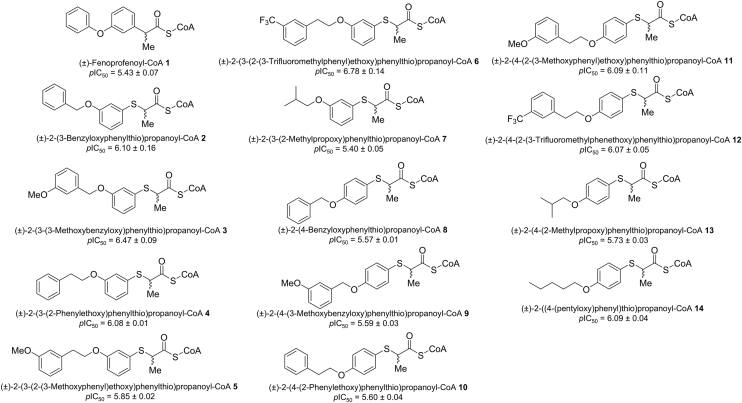
**Chemical structures and *p*IC_50_ values (mean ± SEM, n = 3) of 2-arylthiopropanoyl-CoA inhibitors used in the present study.** Inhibitors were analyzed using dose-response curves (36, 37). The *p*IC_50_ for ±-fenoprofenoyl-CoA **1** is 5.43 ± 0.07 (17).

## Results

### Overall structure of MCR in complex with 2-arylthiopropanoyl-CoA inhibitors

Crystals of wild-type MCR were obtained in 4 M ammonium acetate and 0.1 M Bis-Tris propane (pH 7.0) and soaked with 0.16 to 1.12 mM 2-arylthiopropanoyl-CoA inhibitors for 24 h. All soaked crystals belonged to the *I422* space group, contained six homodimers per asymmetric unit (12 monomers total), with unit cell dimensions isomorphous with the apo-MCR structure (RCSB Protein Data Bank (PDB) ID: 9I2T) (17). Structures were determined between 2.00 to 2.30 Å (selection based on CC_1/2_ and data completeness statistics at the appropriate highest resolution bin) (Table 1).

**Table 1:** X-ray crystallographic data collection and refinement statistics

Crystallographic parameter	Compound 2	Compound 3	Compound 4	Compound 5	Compound 6	Compound 7	Compound 8
Beamline	I04	I04	I04	I04	I04	I04	I04
Wavelength (Å)	0.9537	0.9537	0.9537	0.9537	0.9537	0.9537	0.9537
Crystallographic statistics							
Space group	I422	I422	I422	I422	I422	I422	I422
Unit-cell dimensions							
a, b, c (Å)	276.13 276.13 392.06	276.28 276.28 388.79	277.52 276.52 390.38	276.67 276.67 389.56	276.62 276.62 390.75	277.27 277.27 388.97	277.43 277.43 388.43
α, β, ϒ (°)	90.00 90.00 90.00	90.00 90.00 90.00	90.00 90.00 90.00	90.000 90.000 90.000	90.000 90.000 90.000	90.000 90.000 90.000	90.00 90.00 90.00
Resolution range (Å)^a^	225.76 (2.00)	225.21 (2.20)	226.19 (2.10)	225.57 (2.00)	225.77 (2.25)	225.78 (2.25)	225.76 (2.15)
R_merge_ (Overall and inner)^a^	0.266 (4.657)	0.314 (4.045)	0.319 (4.722)	0.250 (4.601)	0.291 (4.553)	0.316 (4.659)	0.278 (4.267)
R_pim_^a^	0.052 (0.914)	0.061 (0.771)	0.062 (0.953)	0.048 (0.929)	0.056 (0.865)	0.061 (0.941)	0.054 (0.813)
CC_1/2_^a^	0.998 (0.386)	0.997 (0.473)	0.996 (0.630)	0.999 (0.326)	0.997 (0.476)	0.997 (0.580)	0.998 (0.469)
Mean < I/σ(I) >^a^	10.3 (0.9)	9.0 (1.0)	8.4 (0.7)	10.8 (0.8)	9.7 (0.8)	8.9 (0.8)	9.4 (0.8)
Completeness (%)^a^	100.0 (100.0)	100.0 (100.0)	100.0 (100.0)	100.0 (100.0)	100.0 (100.00)	100.0 (100.0)	100.0 (100.0)
No. of observed reflection^a^	13,684,699 (660,013)	10,357,946 (528,355)	11,887,839 (546,011)	13,769,964 (618,757)	9,759,084 (493,806)	9,670,039 (442,555)	11,189,098 (565,439)
No. of unique reflections^a^	500,145 (24,622)	373,895 (18,398)	435,005 (21,421)	498,887 (24,539)	352,301 (17,302)	352,323 (17,298)	403,283 (19,816)
Multiplicity^a^	27.4 (26.8)	27.7 (28.7)	27.3 (25.5)	27.6 (25.2)	27.4 (28.5)	27.4 (25.6)	27.7 (28.5)
Refinement statistics							
R_work_/R_free_	0.22/0.25	0.20/0.24	0.23/0.26	0.21/0.24	0.21/0.25	0.22/0.25	0.22/0.26
RMS deviation in bond lengths (Å)	0.0098	0.0090	0.0093	0.0093	0.0086	0.0090	0.0090
RMS deviation in bond angles (°)	1.61	1.52	1.55	1.56	1.49	1.55	1.53
Ramachandran plot statistics (%)							
Favored	94.09	93.73	94.27	94.30	92.99	94.06	94.01
Allowed	4.90	5.40	4.89	4.67	6.00	4.91	4.91
Outliers	1.01	0.87	0.84	1.03	1.01	1.03	1.08
Average B-Factors (Å^2^)							
Protein	42.7	46.0	42.3	45.2	54.9	47.1	47.3
Ligand	49.6	52.7	51.6	56.2	70.9	57.2	62.0
Water	36.6	37.5	40.6	41.2	42.5	36.2	37.1
No. Atoms							
Protein	32,493	32,541	32,603	32,613	32,547	32,602	32,540
Ligand	804	828	748	840	864	768	804
Water	1291	1393	1858	1562	839	1016	1050


Crystallographic parameter	Compound 9	Compound 10	Compound 11	Compound 12	Compound 13	Compound 14
Beamline	I04	I04	I04	I04	I04	I04
Wavelength (Å)	0.9537	0.9537	0.9537	0.9537	0.9537	0.9537
Crystallographic statistics						
Space group	I422	I422	I422	I422	I422	I422
Unit-cell dimensions						
a, b, c (Å)	276.85, 276.85 390.05	276.86 276.86 389.85	277.17 276.17 391.17	276.77 276.77 390.02	277.19 277.19 388.55	276.58 276.58 389.50
α, β, ϒ (°)	90.00 90.00 90.00	90.00 90.00 90.00	90.000 90.000 90.000	90.00 90.00 90.00	90.000 90.000 90.000	90.00 90.00 90.00
Resolution range (Å)^a^	225.76 (2.30)	225.73 (2.30)	226.15 (2.20)	225.72 (2.20)	225.65 (2.20)	225.51 (2.30)
R_merge_ (Overall and inner)^a^	0.348 (5.549)	0.377 (4.730)	0.308 (5.192)	0.334 (4.078)	0.324 (4.210)	0.342 (4.359)
R_pim_^a^	0.067 (1.054)	0.073 (0.897)	0.060 (1.001)	0.064 (0.798)	0.062 (0.825)	0.066 (0.825)
CC_1/2_^a^	0.996 (0.368)	0.996 (0.423)	0.997 (0.351)	0.998 (0.396)	0.997 (0.391)	0.997 (0.414)
Mean < I/σ(I) >^a^	7.3 (0.7)	8.5 (0.8)	10.8 (0.8)	8.9 (0.9)	9.7 (0.9)	9.4 (0.9)
Completeness (%)^a^	100.0 (100.0)	100.0 (100.0)	100.0 (100.0)	100.0 (100.0)	100.0 (100.0)	100.0 (100.0)
No. of observed reflections^a^	9,086,827 (465,707)	9,121,448 (465,907)	10,368,192 (516,787)	10,447,196 (500,761)	10,372,927 (494,677)	9,143,090 (465,653)
No. of unique reflections^a^	329,974 (16,253)	329,823 (16,226)	378,534 (18,570)	376,404 (18,533)	376,085 (18,496)	328,847 (16,164)
Multiplicity^a^	27.5 (28.7)	27.7 (28.7)	27.4 (27.8)	27.8 (27.0)	27.6 (26.7)	27.8 (28.8)
Refinement statistics						
R_work_/R_free_	0.20/0.24	0.20/0.24	0.21/0.25	0.21/0.25	0.20/0.24	0.21/0.25
RMS deviation in bond lengths (Å)	0.0088	0.0086	0.0089	0.0088	0.0090	0.0089
RMS deviation in bond angles (°)	1.52	1.50	1.52	1.51	1.54	1.67
Ramachandran plot statistics (%)						
Favored	93.15	93.46	93.86	93.74	94.48	93.37
Allowed	5.79	5.58	5.36	5.34	4.75	5.86
Outliers	1.06	0.96	0.78	0.92	0.77	0.77
Average B-Factors (Å^2^)						
Protein	54.7	49.2	48.1	47.0	44.2	50.4
Ligand	73.8	63.0	64.7	69.4	53.7	61.5
Water	44.0	39.1	38.1	35.6	34.4	37.1
No. of atoms						
Protein	32,509	32,626	32,508	32,515	32,608	32,576
Ligand	828	816	840	864	768	780
Water	1144	1296	1099	884	1118	743

a Values in parentheses are for the last-resolution shell.

**Figure 3:**
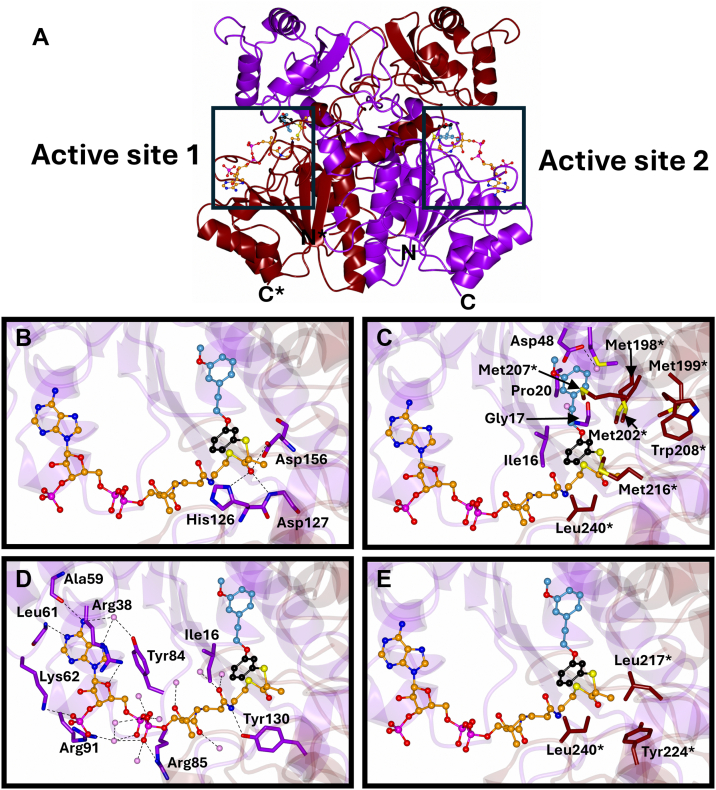
**Structure of MCR dimer with active sites and the side-chains that make up each of the 4 sub-pockets.***A*, the MCR homodimer (subunit/chain 1-*purple* and subunit/chain 2–*tan*) and the 2 active sites it contains. The 2-arylthiopropanoyl-CoA ligand compound **5** (*dark orange*) is shown bound to each active site. The MCR active sites and the residues that make up each of the 4 sub-pockets are shown. *B*, the thioester oxygen binding pocket. *C*, the side-chain binding pocket. *D*, the CoA binding pocket. *E*, the C_α_-methyl group. The 2-arylthiopropanoyl-CoA side-chain group is made of a sulfur-benzene core (shown in *black*) and a variable side-chain (shown in *light blue*). All 2-arylthiopropanoyl-CoAs have the *S*-benzene core in a similar position and near the same side-chains in the MCR side-chain binding pocket. The difference in the side chain and the carbon it is attached to on this *S*-benzene core distinguishes the different 2-arylthiopropanoyl-CoA inhibitors. MCR, α-methylacyl-CoA racemase from *M. tuberculosis*.

Structural alignment with the apo form yielded RMSDs ranging from 0.29 to 0.52 Å across 357 C_α_ atoms, indicating minimal structural variation upon ligand binding (Fig. 3). Each monomer consists of a large domain (Met1-Ala188 and Arg331-Gly360) containing a Rossmann-like fold, and a small domain (Val189-Pro300) with a three-stranded β-sheet. The catalytic residues [His126 (helix 5), Asp156 (helix 6), and Glu241 (helix 9)] are positioned at the domain interface, with Glu241 contributed by the second subunit in the homodimer. These observations are consistent with previous reports (13, 17).

### Active site and ligand binding

Clear and continuous electron density was observed for all 12 active sites in each copy of the structure within the asymmetric unit. Ligand fitting was guided by Fourier difference maps, with ligand B-factors ranging from 68.4 to 119.4 Å^2^. Structures contained between 743 and 1858 water molecules, with higher-resolution structures showing a greater number of observed water molecules.

All ligands were refined as racemic 1:1 mixtures of *R*- and *S-*epimers. The C_α_-H of the *S*-epimer points toward His126; in the *R*-epimer, it points toward Asp156. These conformational differences induce epimer-specific ring rotations of the *S*-benzene core, consistent with racemization.

### Coenzyme A (CoA) binding

The CoA moiety binds in a conserved pocket formed by the large domain. It forms direct contacts with nine residues, including salt bridges with Lys62, Arg85, and Arg91, and π-stacking between Arg38 and the adenine ring. Ile16 rotates to stabilize the thioester sulfur. Water-mediated interactions with five additional residues support the CoA orientation. The B-factors for CoA range from 45.7 to 62.3 Å^2^, with consistent positioning across all structures. These structures are consistent with those previously reported (13, 17).

### Thioester binding pocket

The thioester carbonyl oxygen is stabilized by the main-chain nitrogen of Asp127 and the side-chains of Asp156 (Oδ1 and Oδ2) and His126 (Nδ1). These interactions are supported by Asp127 and two conserved water molecules. Bond distances range from: 2.8 to 3.0 Å (Asp127 N), 2.80 to 3.2 Å (Asp156 Oδ2), 2.7 to 3.1 Å (Asp156 Oδ1), and 3.1 to 3.4 Å (His126 Nδ1). These distances are comparable to those observed in other acyl-CoA complexes (13, 17).

### C_α_-methyl group binding

The C_α_-methyl group resides in a hydrophobic pocket formed by His126, Asp127, Asp156, and Asn152 from one subunit, and Leu217∗, Tyr224∗ and Ile240∗ from the partner subunit. Interatomic distances range from 3.09 to 4.80 Å, with corresponding B-factors of 38.4 to 55.0 Å^2^.

### Side-chain binding pocket

2-Arylthiopropanoyl-CoA inhibitors are based on the structure of ±-fenoprofenoyl-CoA **1** (Fig. 2) (23) and the side-chain group of these inhibitors have a similar core structure in which a *S*-benzene ring is linked to a chiral C_α_ through a sulfur atom (Fig. 2). A diverse peripheral side chain is attached to the *S*-benzene ring. The differences in this peripheral side chain and the angle at which it emerges from the first ring (which in turn is dependent on the *S*-benzene ring substitution pattern) results in structural differences observed in the 13 2-arylthiopropanoyl-CoA crystal structures.

**Figure 4:**
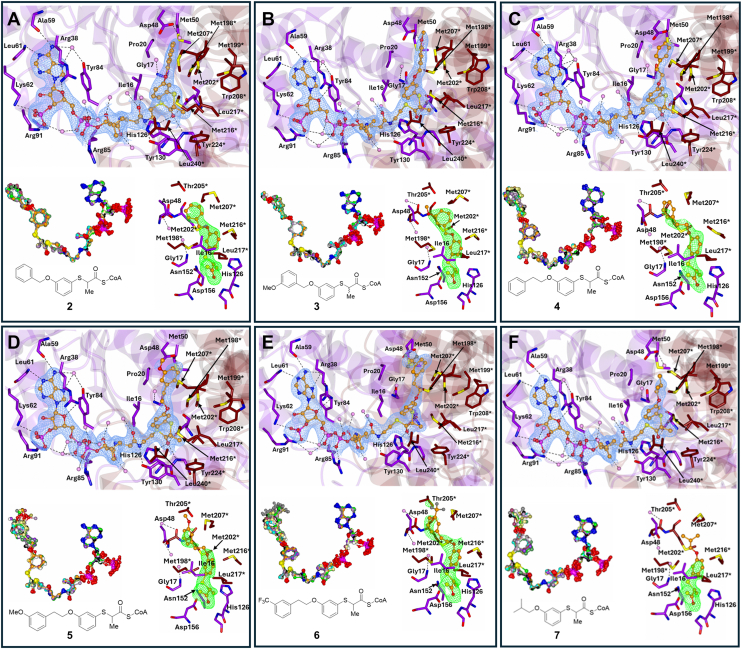
**Binding of 2-arylthiopropanoyl-CoA inhibitors at the MCR active site.** Panels *A–F* show images for compounds **2** to **7**. In each panel (*top*)—portion of the 2Fo-Fc electron density map contoured to 1σ level. Residues from the large domain are in *purple* and those from the small domain are in *tan,* water molecules are in *pink*, and hydrogen bond and ionic interactions are shown as *dashes;* (*bottom left*) -The 12 superimposed ligands in the asymmetric unit and (*bottom right*) - Ligand density (from the unbiased Fo-Fc electron density map contoured at 3σ level). The Fo-Fc electron density map was achieved through PHASER (in molecular replacement) without the ligand and water molecules. The superimposed ligands are colored by atoms with noncarbon atoms in the same color. The electron density maps represent the best ordered site in the crystallographic asymmetric unit. The Fo-Fc maps are close-ups of the 3-substituents. MCR, α-methylacyl-CoA racemase from *M. tuberculosis*.

**Figure 5:**
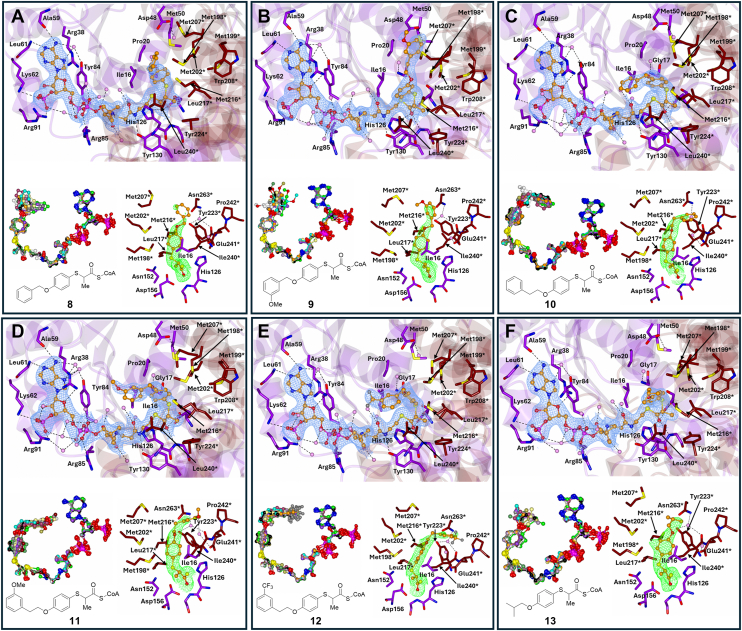
**Binding of 2-arylthiopropanoyl-CoA inhibitors at the MCR active site.** Panels *A*–*F* show images for compounds **8** to **13**. In each panel (*top*)—portion of the 2Fo-Fc electron density map contoured to 1σ. Residues from the large domain are in *purple* and those from the small domain are in *tan*, water molecules are in *pink*, and hydrogen bond and ionic interactions are shown as *dashes*; (*bottom left*)—the 12 superimposed ligands in the asymmetric unit and (*bottom right*)—Ligand density (from the unbiased Fo-Fc electron density map contoured at 3σ). The Fo-Fc electron density map was achieved through PHASER (in molecular replacement) without the ligand and water molecules. The superimposed ligands are colored by atoms with noncarbon atoms in the same color. The electron density maps represent the best ordered site in the crystallographic asymmetric unit. The Fo-Fc maps are close-ups of the 4-substituents. MCR, α-methylacyl-CoA racemase from *M. tuberculosis.*

**Figure 6:**
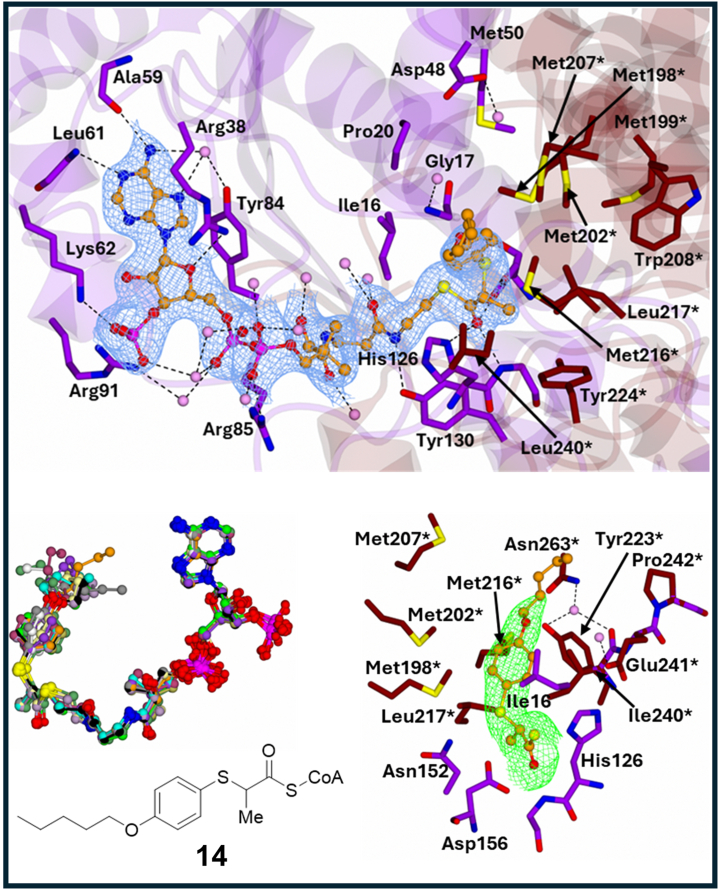
**Binding of 2-arylthiopropanoyl-CoA inhibitor 14 at the MCR active site.** Panel (*top*)—portion of the 2Fo-Fc electron density map contoured to 1σ. Residues from the large domain are in *purple* and those from the small domain are in *tan*, water molecules are in *pink*, and hydrogen bond and ionic interactions are shown as *dashes*; (*bottom left*)—The 12 superimposed ligands in the asymmetric unit and (*bottom right*)—Ligand density (from the unbiased Fo-Fc electron density map contoured at 3σ). The Fo-Fc electron density map was achieved through PHASER (in molecular replacement) without the ligand and water molecules. The superimposed ligands are colored by atoms with noncarbon atoms in the same color. The electron density maps represent the best ordered site in the crystallographic asymmetric unit. The Fo-Fc maps is a close-up of the 4-substituent. MCR, α-methylacyl-CoA racemase from *M. tuberculosis*.

The compound side chains are accommodated in a hydrophobic cavity defined by residues from the Leu195-Tyr223 loop and nearby helices, including Ile16, Met198∗, Met216∗, Trp208∗, Leu240∗, and others. In 8 of 13 structures (*e.g.*, with compounds **2**, **3**, **4**, and **8**), the *S*-benzene core adopts a conserved orientation with limited conformational variability and “moderate” B-factors range from 50 to 90 Å^2^. In the remaining five structures (*e.g.*, **10**, and **11**), rotation around the *S*-benzene bond results in torsion angles between −160.6° and 137.3°, correlating with “high” B-factors ∼75 Å^2^. Based on the active site details within the asymmetric unit for each of the 13 structures, the peripheral 2-arylthiopropanoyl-CoA side-chain group of the different inhibitors occupies only one of the two subpockets. Among these 13 structures, six structures (compounds **2**–**7**) were in the Asp156 side while seven structures (compounds **8**–**14**) were in the His126 side (Table 2) (Figure 4, Figure 5, Figure 6).

**Table 2:** The mean B-factors of 2-arylthiopropanoyl-CoA side-chain group coupled with the *S*-benzene core and peripheral group that make it up

Asp156 sub binding pocket				His126 sub binding pocket			
Compound	Side-chain group Å^2^	*S*-benzene core Å^2^	Peripheral group B-factor Å^2^	Compound	Side-chain group Å^2^	*S*-benzene core Å^2^	Peripheral group B-factor Å^2^
**2**	68.4	53.1	81.8	**8**	99.8	76.1	120.6
**3**	64.3	51.4	73.4	**9**	119.4	91.5	139.3
**4**	75.7	52.8	93.6	**10**	99.2	77.2	116.4
**5**	80.8	56.9	96.1	**11**	106.4	74.1	126.9
**6**	106.3	76.7	122.2	**12**	114.5	72.2	137.2
**7**	91.5	75.4	114.1	**13**	79.1	69.1	93.1
				**14**	89.3	76.9	103.8

The side-chain groups of the 2-arylthiopropanoyl-CoAs **7**, **13**, and **14** do not have a benzene ring in their peripheral side-chain group.

The subpocket boundaries are demarcated by Met207∗ as (i) Asp156 subpocket: smaller, lined by Asp48, Thr205∗, and Met202∗ and (ii) His126 subpocket: larger, lined by Glu241∗, Asn263∗, Ile240∗, and Tyr223∗. Inhibitors occupying the Asp156 subpocket show stable conformations and B-factors in the range of 80 to 120 Å^2^, while those in the His126 subpocket exhibit slight conformational flexibility and higher B-factors (90–140 Å^2^). In all cases, the *S*-benzene core shows lower B-factors than the attached peripheral side-chain group (Table 2).

### Compound 2: peripheral group in Asp156 sub-binding pocket

Compound **2** (Fig. 4*A*) features an *S*-benzene core with an O-CH_2_ linker and a terminal benzene ring. Electron density maps show complete coverage of the side-chain in all active sites, with a B-factor of 68.4 Å^2^. The peripheral group is localized in the Asp156 sub-binding pocket, where it forms stacking interactions with Met202∗ and interacts with residues such as Asp48, Met198∗, Met207∗, and a conserved water molecule. This arrangement is consistent across all active sites.

### Compound 3: O-Me substitution is accommodated by the Asp156 subpocket

Compound **3** (Fig. 4*B***)** features an *S*-benzene ring core with a peripheral group extending from the carbon-3 position. This peripheral group consists of an O-CH_2_ linker, a terminal benzene ring, and a 3-substituted methoxy (O-Me) on the terminal ring.

Electron density maps (2Fo-Fc at 1σ level and Fo-Fc at 3σ level) show strong and consistent coverage of the *S*-benzene core and most of the peripheral group across all active sites. The clearest density appears in sites A, D, F, I, and J, with site H displaying the weakest signal. The overall B-factor of the inhibitor side-chain is 64.3 Å^2^, with values of 51.4 Å^2^ for the core and 73.4 Å^2^ for the peripheral group.

Within the Asp156 pocket, **3** forms stabilizing interactions with Asp48, Thr205, Met207, Met202, Met198, and Met216∗, as well as conserved water molecules coordinated by Asp48 and Arg47. The terminal benzene ring engages in π-stacking with Met202∗ (3.48–3.89 Å), flanked by Met198∗ (5.03–5.54 Å) and Met207∗ (3.25–3.79 Å), which help stabilize the conformation. The O-Me group also contributes to this stabilization: its methyl moiety points toward a conserved water near Arg47 (4.21–5.10 Å), while the methoxy oxygen interacts with a water molecule near Asp48 (3.41–4.31 Å), enabling potential hydrogen bonding.

### Compound 4: extended connector and variable conformations

Compound **4** (Fig. 4*C*), similar to **2**, has an additional CH_2_ group in the connector between the *S*-benzene core and terminal benzene ring. Electron density maps show full coverage of the *S*-benzene core and most of the peripheral group in all active sites, with a B-factor of 75.7 Å^2^. The peripheral group interacts with Met207∗, Asp48, Met202∗, and Thr205∗, adopting two distinct conformations: one with the terminal benzene ring interacting with Met202∗, and another where the terminal ring stacks with Met207∗.

### Compound 5: methoxy group modulates binding conformation

Compound **5** (Fig. 4*D*) has a similar structure to 4 but with a methoxy (O-Me) group at the C3 position of the terminal benzene ring. Electron density maps show partial coverage of the O-Me group, with a B-factor of 80.8 Å^2^. The peripheral group extends into the Asp156 sub-binding pocket, interacting with Met207∗, Arg47, and Met202∗. Two conformations are observed: one with the terminal benzene ring stacking with Met202∗, and another where the terminal ring interacts with Met207∗.

### Compound 6: CF_3_ substituent alters binding stability

Compound **6** (Fig. 4*E*) features an additional CF_3_ group at the C3 position of the terminal benzene ring of the inhibitor. Electron density maps show almost complete coverage of the side-chain, with a B-factor of 106.3 Å^2^. The peripheral group occupies the Asp156 sub-binding pocket and interacts with residues such as Met207∗, Arg47, Met202∗, and Thr205∗. Two distinct orientations of the terminal benzene ring are observed: one with the CF_3_ group facing Ser46 and Arg47, and the other with the CF_3_ group pointing toward Thr205∗.

### Compound 7: dual conformations in Asp156 sub-binding pocket

Compound **7** (Fig. 4*F*) features an *S*-benzene core with an O-CH_2_ linker and a CH_3_-CH-CH_3_ terminal group, similar to **13**. Electron density maps show partial coverage of the side-chain, with the terminal group resolved in sites A, D, F, J, and L. The B-factor for the side-chain is 91.5 Å^2^, with B-factors for the *S*-benzene core and peripheral group at 75.4 Å^2^ and 114.1 Å^2^, respectively. The peripheral group forms two conformations in the Asp156 sub-binding pocket: one where the O-CH_2_ linker interacts with a water molecule associated with Gly17, and another where the terminal CH_3_-CH-CH_3_ group is near Met198∗, Met207∗, and Met202∗.

### Compound 8: S-benzene core with O-CH_2_ linker and terminal benzene ring

Compound **8** (Fig. 5*A*) features a sulfur-linked benzene core with a peripheral group comprising an O-CH_2_ connector and a terminal benzene ring. The 2Fo-Fc (1σ) and Fo-Fc (3σ) electron density maps show consistent coverage of the *S*-benzene core across all 12 active sites, while the peripheral group exhibits partial density in most sites, particularly molecules within the asymmetric unit A, D, F, and J. Notably, sites E, H, and I lack significant peripheral density. The side chain extends toward the His126 sub-binding pocket, interacting with residues including Ile240∗, Met216∗, Met207∗, and Asn263, as well as a conserved water molecule. In the predominant conformation, the terminal benzene ring engages in π-stacking with Met216∗ and is positioned near the Ile240 and Asn263 residues, along with the conserved water molecule. The side chain exhibits a B-factor of 99.8 Å^2^, with the *S*-benzene core and peripheral group at 76.1 and 120.6 Å^2^, respectively.

### Compound 9: loss of aromatic stacking due to O-Me group

Compound **9** (Fig. 5*B*) shares a similar core and peripheral structure to **8** but incorporates an O-Me group at the 3-position of the terminal benzene ring. The electron density maps (2Fo-Fc at 1σ and Fo-Fc at 3σ) show coverage of the *S*-benzene core in all sites and partial coverage of the terminal benzene in sites A, D, F, and G. However, the O-Me group was not resolved in any site. The side chain maintains a B-factor of 119.4 Å^2^, with the peripheral group showing a higher B factor (139.3 Å^2^) compared to **8**. The side chain occupies the His126 subpocket, with interactions near Met207∗, Ile240∗, Met216∗, and Asn263∗. Unlike 8, π-stacking with Met216∗ is absent, likely due to steric interference from the O-Me group.

### Compound 10: extended O-CH_2_-CH_2_ connector alters peripheral group orientation

Compound **10** (Fig. 5*C*) introduces an O-CH_2_-CH_2_ connector between the *S*-benzene core and terminal benzene ring. Electron density covers the *S*-benzene core in all sites, with partial coverage of the terminal ring in sites A, B, D, F, and K. The B-factor for the side-chain is 99.2 Å^2^, with the peripheral group exhibiting a B-factor of 116.4 Å^2^. In 11 of 12 active sites, the peripheral group adopts a consistent conformation, interacting with the His126 subpocket near Ile240, Glu241∗, and Pro242∗. The terminal benzene participates in π-stacking with this triad (3.69–5.84 Å), and the longer connector shifts the peripheral group's orientation away from Met216∗ and toward the Ile240-Glu241∗-Pro242∗ cluster.

### Compound 11: O-Me group stabilizes peripheral orientation *via* water mediated interaction

Compound **11** (Fig. 5*D*) shares structural features with **10** and **12**, with an O-CH_2_-CH_2_ linker and a terminal benzene ring but incorporates an O-Me group at the 3-position. Electron density maps show full coverage of the *S*-benzene core and connector in all sites, with the terminal ring and O-Me group fully resolved only in sites D, F, and I. The side-chain's B-factor is 106.4 Å^2^, with the peripheral group at 126.9 Å^2^. In all sites, the side-chain extends into the His126 subpocket, with the terminal benzene ring forming π-stacking interactions with the Ile240-Glu241∗-Pro242∗ triad (3.62–4.71 Å) and interacting with conserved waters. The O-Me group forms stabilizing hydrogen bonds with the Glu241∗-associated water (3.09–3.61 Å), reducing the conformational flexibility observed in **11**.

### Compound 12: CF_3_ group impairs peripheral group stability

Compound **12** (Fig. 5*E*), similar to 10, features an O-CH_2_-CH_2_ linkage and a terminal benzene but with a CF_3_ substituent at the 3-position. Electron density maps show coverage of the *S*-benzene core and connector in all sites, but partial density is observed for the terminal group only in sites D, F, and I. The side-chain's B-factor is 114.5 Å^2^, and the CF_3_ group exhibits a B-factor of 140.2 Å^2^, indicating reduced conformational stability. The side-chain binds to the His126 sub-pocket in all sites, near Ile240, Glu241∗, and Pro242∗, with conserved waters on either side. In sites D, F, and I, the terminal benzene stacks with the Ile240-Glu241∗-Pro242∗ triad, but the CF_3_ group disrupts consistent binding, as evidenced by the limited density in most sites compared to compound **10**.

### Compound 13: peripheral group with CH_3_-CH-CH_3_ terminal group

Compound **13** (Fig. 5*F*) features an *S*-benzene core with an O-CH_2_ linker and a terminal CH_3_-CH-CH_3_ group. Electron density maps show clear coverage of the *S*-benzene core and part of the peripheral group in all active sites, except E, H, and L, where only the connector group is resolved. The B-factor for the side-chain is 79.1 Å^2^, with the *S*-benzene core and peripheral group showing B-factors of 69.05 Å^2^ and 93.1 Å^2^, respectively. In the 12 active sites, the peripheral group interacts with residues near Met207∗, Met216∗, Ile240∗, Asn263∗, and a conserved water molecule. The terminal CH_3_-CH-CH_3_ group stacks with Met216∗, restricting its mobility.

### Compound 14: extended O-CH_2_-CH_2_-CH_2_ chain with stacking interaction

Compound **14** (Fig. 6*A*) is similar to **13** but has a longer peripheral group consisting of an O-alkyl chain. Electron density maps reveal strong coverage for the *S*-benzene core and most of the peripheral group, with the weakest coverage in site H and the strongest in site F. The B-factor for the side-chain is 89.3 Å^2^, with B-factors for the *S*-benzene core and peripheral group at 76.9 Å^2^ and 103.8 Å^2^, respectively. The peripheral group extends into the His126 sub-binding pocket, interacting with Met207∗, Met216∗, Ile240∗, Asn263∗, and a conserved water molecule. The terminal CH_2_-CH_2_-CH_3_ group forms a stacking interaction with Met216∗, similarly to that observed for compound **13**.

### MCR inhibition by 2-arylthiopropanoyl-CoA ligands

**Figure 7:**
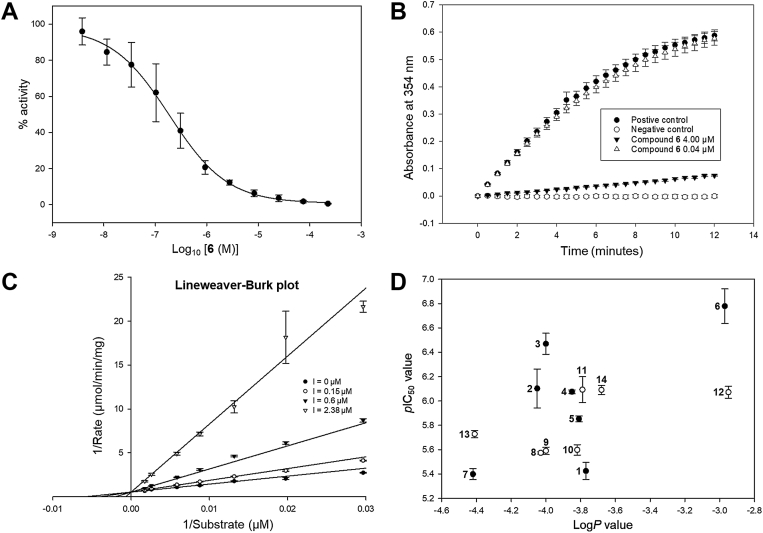
**Inhibition of MCR activity by 2-arylthiopropanoyl-CoAs, as measured by the colorimetric assay.***A*, a representative dose-response curve for compound **6**. Data are mean ± SD for two technical repeats. *B*, a jump dilution experiment showing reversible inhibition of MCR by compound **6**. Data are mean ± SD for three technical repeats. *C*, a Lineweaver-Burk plot showing competitive inhibition of MCR activity by compound **6**. Data are mean ± SD for three technical repeats for a representative independent repeat. *D*, a plot of *p*IC_50_ values taken from Figure 1 against Log*P* values [taken from (23)]. *Closed black circles* are for compounds with 3-substituted side-chains; *open white circles* are for compounds with 4-substituted side-chains. Data are mean ± SEM (n = 3). MCR, α-methylacyl-CoA racemase from *M. tuberculosis*.

The inhibitory potency of thirteen 2-arylthiopropanoyl-CoAs was measured *via* a colorimetric assay (35), and the *p*IC_50_ values were extrapolated from dose-response curves (36, 37). These ranged between 5.40 to 6.78 (Table 3, Figs. 7*A* and S2–S15), corresponding to a range of potencies between 3.98 and 0.17 μM, respectively. Among these, compound **6** (*p*IC_50_ = 6.78 ± 0.14) emerged as the most potent inhibitor, while compound **7** (*p*IC_50_ = 5.40 ± 0.05) was the least potent (Figs. 2 and 7). Compound **7** has similar potency to fenoprofenoyl-CoA 1 (*p*IC_50_ of 5.43 ± 0.07) (Table 3). The *p*IC_50_ of **6** indicates that it is at least 13.5 times more potent than ibuprofenoyl-CoA (*p*IC_50_ = 5.65 ± 0.05) and analogs previously reported (17), and even more potent than the six straight-chain CoA esters in the same study. All compounds were reversible inhibitors (Figs. 7*B* and S15).

**Table 3:** *p*IC_50_ values of 2-arylthiopropanoyl-CoA inhibitors studied

Peripheral group emerges from the Carbon-3 of the *S*-benzene core			Peripheral group emerges from the Carbon-4 of the *S*-benzene core		
Compound	Calculated Log*P*	*p*IC_50_	Compound	Calculated Log*P*	*p*IC_50_
**2**	−4.05	6.10 ± 0.16	**8**	−4.03	5.57 ± 0.01
**3**	−4.00	6.47 ± 0.09	**9**	−4.00	5.59 ± 0.03
**4**	−3.85	6.08 ± 0.01	**10**	−3.82	5.60 ± 0.04
**5**	−3.81	5.85 ± 0.02	**11**	−3.79	6.09 ± 0.11
**6**	−2.97	6.78 ± 0.14	**12**	−2.95	6.07 ± 0.05
**7**	−4.42	5.40 ± 0.05	**13**	−4.41	5.73 ± 0.03
			**14**	−3.68	6.09 ± 0.04

Log*P* values are taken from reference ^23^.

Kinetic analysis of **6** (Figs. 7 and S16) demonstrated competitive inhibition. The *K*_i_ value for **6** was determined as 0.238 ± 0.039 μM (mean ± SEM, n = 3). This compares to 3.04 ± 0.34 μM (mean ± SEM, n = 3) for ibuprofenoyl-CoA (17). Thus, **6** is around 13 ⨯ more potent than ibuprofenoyl-CoA, as judged by *K*_i_ values.

The comparison of *p*IC_50_ values for the 2-arylthiopropanoyl-CoA inhibitors with those of ±-fenoprofenoyl-CoA **1** reveals that the 2-arylthiopropanoyl-CoAs are more potent, with inhibitory potencies ranging from 22 to 0.94 times. This suggests that the 2-arylthiopropanoyl-CoAs represent an iterative improvement over the parent ±-fenoprofenoyl-CoA **1**, likely due to facile enolate formation. This improvement can be clearly seen as ±-fenoprofenoyl-CoA **1** is less potent than 3-substituted 2-arylthiopropanoyl-CoAs with similar lipophilicity (Fig. 7). The crystal structures further support this observation, with the improvement in potency likely attributable to the unique *S*-benzene scaffold present in the side-chain group, which promotes facile enolate intermediate formation. 2-Arylthiopropanoyl-CoAs such as **7** (*p*IC_50_ = 5.40 ± 0.05), **13** (*p*IC_50_ = 5.73 ± 0.03), and **14** (*p*IC_50_ = 6.09 ± 0.04) were found to be more potent than branched-chain acyl-CoA esters, such as 2-methyldecanoyl-CoA (17). Inhibitor potency correlates with compound lipophilicity and the substitution pattern of the S-benzene ring (Fig. 7*D*).

## Discussion

### Implications of 2-arylthiopropanoyl-CoA inhibitor binding on MCR catalytic mechanism

2-Arylthiopropanoyl-CoA inhibitors, specifically 2-methylacyl-CoA esters derived from ±-fenoprofenoyl-CoA **1**, are expected to undergo a 1:1 proton transfer reaction, similar to the behavior observed with 2-methylacyl-CoA esters (17). This process involves the deprotonation of the C_α_-H of either the *S*- or *R*-epimer by His126 or Asp156, respectively, forming an enolate intermediate (18, 20), which is then reprotonated nonstereoselectively to produce a near 1:1 ratio of the product epimers (20, 21).

The MCR structure reveals four distinct binding pockets: the acyl-CoA ester, thioester oxygen, C_α_-methyl group, and the side-chain binding pocket, all of which are consistent with previous reports (12, 13, 17). Each of these binding pockets is appropriately occupied by the 2-arylthiopropanoyl-CoA inhibitors. Within the active site, a subtle clockwise or counterclockwise rotation of the α-carbon is observed following the 1,1-proton transfer reaction, with the side-chain binding group held in a stationary position. This mechanism aligns with that proposed by Mojanaga *et al.* (17) but contradicts the mechanism suggested by Bhaumik *et al.* (13) which reported that epimerization occurs through the movement of the side chain across a methionine-rich region.

Bhaumik *et al.* (13) had previously proposed a mechanism in which the side-chain binding site was divided into separate *R*- and *S*-epimer regions, extrapolated from MCR crystal structures of (2*S*)-methylmyristoyl-CoA, (2*R*)-methylmyristoyl-CoA, and ibuprofenoyl-CoA. However, our results (17) diverge from this model. The crystal structures of 2-arylthiopropanoyl-CoA inhibitors suggest the side-chain binding site comprises two sub-binding pockets delineated by a Met207∗ side-chain, consistent with the observations of Bhaumik *et al.* (13). However, our findings indicate that these two regions are not stereospecific for the *R*- or *S*-epimers, as previously suggested. Instead, the occupied sub-binding pocket is determined by the substitution pattern of the side-chain group, and epimerization does not result in a shift in the binding pocket.

### Effect of structural changes in 2-arylthiopropanoyl-CoA side-chain groups

The *S*-benzene core—All 13 2-arylthiopropanoyl-CoA inhibitors share a rigid *S*-benzene core in their side-chain group, which is bound within the MCR side-chain binding pocket. Structural analysis revealed that while some structures exhibited limited torsion angle mobility, the B-factors of the side-chain *S*-benzene core (52.8–91.5 Å^2^) were consistently lower than those of the peripheral groups (81.8–137.2 Å^2^). This suggests that the *S*-benzene core is crucial to the inhibitor's potency, serving as an effective scaffold for inhibitor design.

Peripheral group positioning: carbon-4 *versus* carbon-3 on the S-benzene core—Significant differences in *p*IC_50_ values were observed between 2-arylthiopropanoyl-CoAs with identical chemical formulas but differing in the position of the peripheral group on the *S*-benzene core. In general, 2-arylthiopropanoyl-CoAs with a peripheral group attached to carbon-3 of the *S*-benzene core exhibited lower IC_50_ values (IC_50_ 0.17–3.99 μM) than those with the peripheral group attached to carbon-4 (IC_50_ 0.81–2.67 μM). This effect is most pronounced with compounds possessing highly lipophilic groups appended to the *S*-benzene core, such as the compound **4**/**10** pair. Compounds with small, nonlipophilic groups appended to the *S*-benzene core, such as the compound **7**/**13** pair, have almost identical potencies (Fig. 2). This trend can be attributed to the different sub-binding pockets within the side-chain binding site. 2-Arylthiopropanoyl-CoAs with side-chains attached to carbon-3 only occupy the smaller Asp156 sub-binding pocket, while 2-arylthiopropanoyl-CoAs with side-chains attached to carbon-4 only occupy the larger His126 sub-binding pocket.

Effect of CF_3_ and O-Me auxiliary groups—The inclusion of auxiliary groups, such as -CF_3_ and O-Me, at the terminal benzene ring of the side-chain group influenced the inhibitory potency of the 2-arylthiopropanoyl-CoAs. The relationship between the substitution pattern in the terminal ring and potency is complex. For a set of compounds with 3-subsitutions appended to the *S*-benzene core, the addition of an O-Me group decreased potency compared to the unsubstituted compound, as seen in the compound **5**/**4** pair (*p*IC_50_ = 5.85 ± 0.02 *versus* 6.08 ± 0.01). But for some pairs, the inclusion of this same O-Me group increased potency compared to the unsubstituted compound, as seen in the compound **2**/**3** pair (*p*IC_50_ = 6.10 ± 0.16 *versus* 6.47 ± 0.09). On the other hand, inclusion of a 3-OMe group on the distal ring of 4-substituted compounds had little effect on potency (**9** *p*IC_50_ = 5.59 ± 0.03 *versus* **8** *p*IC_50_ = 5.57 ± 0.01). Similarly, inclusion of a 2-OMe group in compounds with a longer side-chain also increased potency (**10** *p*IC_50_ = 5.60 ± 0.04 *versus* **11** *p*IC_50_ = 6.09 ± 0.11). Inclusion of a CF_3_ group had a larger effect on potency in 3-substituted compounds (**6**) than in 4-substituted compounds (**12**) compared to compounds lacking the group (**4** and **10**, respectively). The structural analysis suggested that these auxiliary groups improve the interaction between the 2-arylthiopropanoyl-CoA side-chain group and hydrophobic residues within the side-chain binding pocket, possibly through enhancing favorable interactions or introducing steric clashes that disrupt suboptimal binding.

The detailed structural analysis of 2-arylthiopropanoyl-CoA inhibitors highlights how the position and nature of the peripheral group relative to the *S*-benzene core affects the binding conformation and interactions within specific sub-binding pockets of the MCR enzyme. In the 4-substituted compounds which exclusively occupy the His126 sub-binding pocket, compounds with shorter connectors (*e.g.*, **8** and **9**) interact more with Met216∗, while those with longer connectors (*e.g.*, **10**, **11**, and **12**) interact more with the Ile240-Glu241∗-Pro242∗ triad. Conversely 3-substituted compounds in Asp156 sub-binding pocket with shorter connectors (*e.g.*, compounds **2** and **3**) interact with only Met202∗ while those with longer connectors (*e.g.*, compounds **4**, **5**, and **6**) interact more with both Met202∗ and Met207∗. Variations in terminal groups, such as -CF_3_ or O-Me, modulate binding stability by inducing steric clashes or forming stabilizing hydrogen bonds, providing key insights for designing dual-pocket inhibitors.

## Conclusion

Cholesterol is an important energy source for growth and persistence in *Mtb* infection (5, 6), and targeting cholesterol and cholesterol ester metabolism (Fig. 1) is a promising therapeutic strategy. The viability of this approach is shown by genetic knock-down of CYP1251A, which inhibits *Mtb* growth by nutrient deprivation and accumulation of toxic upstream metabolites (7). Small molecule dual CYP125A1 and CYP142A1 inhibitors strongly inhibit *Mtb* growth (38) by blocking the Ω-oxidation of cholesterol and cholesterol esters. Inhibition of cholesterol metabolism using MCR inhibitors requires the formation of *R*-2-methylacyl-CoA intermediates to be effective. The relative importance of CYP125A1 (producing intermediates with 25*S*- configuration) and CYP142A1 (producing intermediates with 25*R*-configuration) during cholesterol metabolism (Fig. 1*A*) is unknown. Cholesterol sulfate and cholesterol propanoate are exclusively metabolized by CYP142A1 (9) to give intermediates with 25*R*-configuration (8) (Fig. 1*B*). Therefore, the success of inhibiting MCR as a strategy is dependent on both the relative importance of cholesterol and cholesterol esters as energy sources for *Mtb*, and the proportion of cholesterol which forms intermediates with 25*R* configuration. If the 25*R*-epimers are formed as the major product of cholesterol (ester) metabolism, then inhibiting MCR is likely to be a highly effective therapeutic strategy. There is potential that FAR will allow *Mtb* to evade the effects of MCR inhibition by converting 25*R*-substrates to their corresponding 25*S*- epimers, thereby enabling β-oxidation. Catalytic activity of FAR has not yet been demonstrated (15), but simultaneous inhibition of MCR and FAR would be required for a potent therapeutic effect if FAR was proven to convert substrates derived from cholesterol.

This study presents a comprehensive analysis of the MCR in complex with 13 2-arylthiopropanoyl-CoA esters, providing insights into their potential as inhibitors of the enzyme. The catalytic mechanism of MCR in this study is consistent with that observed for other acyl-CoA esters, involving deprotonation and non-stereoselective reprotonation of the 2-arylthiopropanoyl-CoA acyl-CoA, which results in epimerization at the C_α_ position with minimal change to the side-chain conformation. This mechanism differs from that proposed by Bhaumik *et al.* (13) who suggested substantial side-chain movement but aligns with the model proposed by Mojanaga *et al.* (17).

The side-chain group 2-arylthiopropanoyl-CoA inhibitors occupy one of two sub-binding pockets in the MCR active site, with the position determined by the structure of the side-chain group. These findings highlight the importance of the *S*-benzene scaffold in inhibitor design, as well as the potential for optimizing inhibitory potency by modifying the side-chain group or incorporating auxiliary groups. The results presented herein and those by Yevglevskis *et al.* (23) on human AMACR imply that it may be possible to inhibit MCR while sparing human AMACR activity upon further optimization of side chains. Derivatives utilizing both 3- and 4- appended side chains are expected to enhance both potency and selectivity. Use of acyl-CoA esters as therapeutic agents presents challenges as they do not comply with Lipinski's guidelines (39, 40) because of their high molecular weight (∼1100 Da.), their highly charged groups, and the large number of hydrogen bond donors and acceptors. This study lays the groundwork for rational inhibitor design based on a prodrug approach (1, 2, 3) utilizing for example, the precursor carboxylic acids (25, 31) which may be converted to the acyl-CoA drug *in vivo*. This study also offers avenues for future drug discovery efforts, such as fragment-based or high-throughput screening approaches incorporating structure-based drug design to develop more druggable small-molecule inhibitors.

## Experimental procedures

Laboratory reagents were obtained from Merck and Thermo Fisher Scientific and used without further purification. Molecular biology reagents were sourced from New England BioLabs, Stratagene, Promega, and Novagen. The pET3a plasmid for wild-type MCR was synthesized by Genewiz. Protein columns and resins were purchased from Cytiva Life Sciences, and crystallization screening tools were sourced from Molecular Dimensions. Colorimetric substrate (35), ±-fenoprofenoyl-CoA **1** (21) and 2-arylpropionyl-CoA esters **2** to **14** (23) were synthesized according to previously published methods. Solutions of substrate and inhibitors were quantified *via* ^1^H NMR using an internal standard as previously described (17). Acyl-CoA stock solutions were vortex-mixed for 5 min and centrifuged at 13.3 kRPM for 3 min before use in crystallization or kinetic experiments. Recombinant *M. tuberculosis* MCR was expressed and purified as previously described (12, 41), buffer-exchanged into 10 mM potassium phosphate pH 8.8, and concentrated to 6 to 7.2 mg/ml before being stored at −80 °C. All other reagents were procured through UK suppliers or agents.

### X-ray crystallography studies

Crystallization was performed using hanging drop vapor diffusion at 22 °C. Crystals of wild-type MCR were grown in a 24 well plate using condition 4 M ammonium acetate and 0.1 M Bis-Tris propane (pH 7.0) [derived from LMB screen well A5 (Molecular Dimensions)]. Each well of the 24-well crystallization plate (Molecular Dimensions) contained 500 μl of reservoir solution, with recombinant wild-type MCR protein mixed with reservoir solution in a 2:1 ratio to make 3 μl drops. Plates were incubated for 5 weeks at 22 °C. Crystals grew to their maximum size after approximately 4 weeks. To obtain MCR in complex with 2-arylthiopropanoyl-CoA inhibitors, wild-type MCR crystals were soaked in 0.16 to 1.12 mM 2-arylthiopropanoyl-CoA inhibitor in reservoir solution for 24 h at 22 °C. Soaked crystals were flash-frozen using liquid nitrogen (100 K) without additional cryoprotectant and data collected at beamline I04, Diamond Light Source. X-ray diffraction images (3600) were collected at 0.1° oscillation, 6.8 to 7.2 ms exposure, 100% transmission, and 10 to 12 MGy dose using the Eiger2 XE 16M detector (Dectris). The highest resolution datasets were indexed and integrated using DIALS and scaled using AIMLESS (CCP4i2 suite). The structure solution was performed *via* molecular replacement in PHASER with a previously reported MCR structure (PDB 9I2T) without the ligand and water molecules to obtain “unbiased” electron density map. For 2-arylthiopropanoyl-CoA complex structures, ligand restraint libraries were built using ACEdrg (42) and added manually to Fourier difference (Fo-Fc) maps in COOT (43, 44). Refinement was carried out with REFMAC5 (45), adding water molecules and validating the refined model using MolProbity (46, 47) and PDB validation (48). All 156 protein chains among the 13 structures were manually inspected to avoid any bias in ligand fitting. Structural illustrations were prepared using CCP4mg (49).

### Inhibition assay

Inhibition assays were conducted as previously described (17) in 50 mM NaH_2_PO_4_-NaOH, 100 mM NaCl, 1 mM EDTA (pH 7.4), and 3% (v/v) dimethylsulfoxide. Product formation was monitored at 354 nm. Assay components at final concentrations included MCR (3.12 nM), inhibitor (3.81 nM to 225 μM), and substrate (96 μM). Enzyme and inhibitor were preincubated for 10 min before adding substrate to initiate the assay. Rates were determined using ICEKAT (50, 51), and dose-response curves (36, 37) were fitted using SigmaPlot 15.0 with a 4-parameter logistic model. Data are reported as *p*IC_50_ ± SEM (n = 3). Reversibility of inhibition was assessed using a jump-dilution experiment (52) over 12 min with enzyme (31.2 nM), substrate (96 μM), and inhibitor at 24 × or 0.24 × IC_50_.

### Inhibition mode determination

Kinetic data were collected using compound **6** (0, 0.15, 0.6, and 2.38 μM) and MCR (3.12 nM) in 50 mM NaH_2_PO_4_-NaOH, 100 mM NaCl, and 1 mM EDTA (pH 7.4), with 3% (v/v) dimethylsulfoxide and varying substrate concentrations (50.2–576 μM). Enzyme and inhibitor solutions were pre-incubated for 10 min before adding these mixtures to the substrate to initiate the assay. Rates were determined using ICEKAT (50, 51) and analyzed with SigmaPlot 15.0 using Simple EK and enzyme kinetics macros (53). The best-fitting inhibition model was selected based on graphical analysis and statistical ranking of models. The *K*_i_ value was determined as the mean ± SEM from three independent repeats.

## Data availability

The atomic coordinates and structure factors for all 13 structures [under accession codes – 9RYY (2-(3-Benzyloxyphenylthio)propanoyl-CoA **2** complex), 9RZA (2-(3-(3-Methoxybenzyloxy)phenylthio)propanoyl-CoA **3** complex), 9RZ6 (2-(3-(2-Phenylethoxy)phenylthio)propanoyl-CoA **4** complex), 9RZ8 (2-(3-(2-(3-Methoxyphenyl)ethoxy)phenylthio)propanoyl-CoA **5** complex), 9RZ7 (2-(3-(2-(3-Trifluoromethylphenyl)ethoxy)phenylthio)propanoyl-CoA **6** complex), 9RZ9 (2-(3-(2-Methylpropoxy)phenylthio)propanoyl-CoA **7** complex), 9RYZ (2-(4-Benzyloxyphenylthio)propanoyl-CoA **8** complex), 9RZ0 (2-(4-(3-Methoxybenzyloxy)phenylthio)propanoyl-CoA **9** complex), 9RZ1 (2-(4-(2-Phenylethoxy)phenylthio)propanoyl-CoA **10** complex), 9RZ4 ((±)-2-(4-(2-(3-Methoxyphenyl)ethoxy)phenylthio)propanoyl-CoA **11** complex), 9RZ2 (2-(4-(2-(3-Trifluoromethylphenethoxy)phenylthio)propanoyl-CoA **12** complex), 9RZ3 (2-(4-(2-Methylpropoxy)phenylthio)propanoyl-CoA **13** complex), 9RZ5 ((±)-2-(4-Pentyloxyphenylthio)propanoyl-CoA **14** complex) respectively] have been deposited in the RCSB Protein Data Bank, www.pdb.org.

## Supporting information

This article contains supporting information.

## Conflict of interest

The authors declare that they have no conflicts of interest with the contents of this article.
