# Supplementary material for: Structural basis for inhibition of Mycobacterium tuberculosis α-methylacyl-CoA racemase by 2-arylthiopropanoyl-CoA inhibitor analogs
Source: J Biol Chem. 2025 Oct 22;301(12):110848. doi: 10.1016/j.jbc.2025.110848 (PMC12682127; doi:10.1016/j.jbc.2025.110848)
Supplement: Supporting Figures [file mmc1.docx]

**Supplementary Information**

**Structural basis for inhibition of *Mycobacterium tuberculosis* α-methylacyl-CoA racemase by 2-arylthiopropanoyl-CoA inhibitor analogs**

**Otsile O. Mojanaga, Timothy J. Woodman, Matthew D. Lloyd*, and K. Ravi Acharya***

*From the University of Bath, Department of Life Sciences, Claverton Down, Bath BA2 7AY, United Kingdom*

*For correspondence: K. Ravi Acharya, [bsskra@bath.ac.uk](mailto:bsskra@bath.ac.uk) or Matthew D. Lloyd, [M.D.Lloyd@bath.ac.uk](mailto:M.D.Lloyd@bath.ac.uk)

MCR -----MAGPLSGLRVVELAGIGPGPHAAMILGDLGADVVRIDRPSSVDGISRDAMLRNR-

FAR ---MTTGGPLAGVKVIELGGIGPGPHAGMVLADLGADVVRVRRPGGLTMPSEDRDLLHR-

Rv3272 MPTSNPAKPLDGFRVLDFTQNVAGPLAGQVLVDLGAEVIKVEAPGGEAARQITSVLPGRP

MCR -------------RIVTADLKSDQGLELALKLIAKADVLIEGYRPGVTERLGLGPEECAK

FAR -------------GKRIVDLDVKTQPQAMLELAAKADVLLDCFRPGTCERLGIGPDDCAS

Rv3272 PLATYFLPNNRGKKSVTVDLTTEQAKQQMLRLADTADVVLEAFRPGTMEKLGLGPDDLRS

MCR VNDRLIYARMTGWGQTGPRSQQAGHDINYISLNGILHAIGRGDERP-VPPLNLVGDFGGG

FAR VNPRLIFARITGWGQDGPLASTAGHDINYLSQTGALAAFGYADRPP-MPPLNLVADFGGG

Rv3272 RNPNLIYARLTAYGGNGPHGSRPGIDLVVAAEAGMTTGMPTPEGKPQIIPFQLVDNASG-

MCR SMFLLVGILAALWERQSSGKGQVVDAAMVDGSSVLIQMMWAMRATGMWTDT---------

FAR SMLVLLGIVVALYERERSGVGQVVDAAMVDGVSVLAQMMWTMKGIGSLRDQ---------

Rv3272 -HVLAQAVLAALLHRERNGVADVVQVAMYDVAVGLQANQLMMHLNRAASDQPKPEPAPKA

MCR RGANMLDGGAPYYDTYECADGRYVAVGAIEPQFYAAMLAGLG-----LDAAELPPQNDRA

FAR RESFLLDGGAPFYRCYETSDGKYMAVGAIEPQFFAALLSGLG-----LSAADVPTQLDVA

Rv3272 KRRKGVGFATQPSDAFRTADG-YIVISAYVPKHWQKLCYLIGRPDLVEDQRFAEQRSRSI

MCR RWPELRALLTEAFASHDRDHWGAVFANSDACVTPVLAFGEVHNEPHIIERNTFYEANGGW

FAR GYPQMYDIFAERFASRTRDEWTRVFAGTDACVTPVLAWSEAANNDHLKARSTVITAHGVQ

Rv3272 NYAELTAELELALASKTATEWVQLLQANGLMACLAHTWKQVVDTPLFAENDLTLEVGRGA

MCR QPMPAP----RFSRTASSQPRPPAATIDIEAVLTDWDG

FAR QAAPAP----RFSRTPAGPVRPPPAAATPIDEINW---

Rv3272 DTITVIRTPARYASFRAVVTDPPPTAGEHNAVFLARP-

**Figure S1:** Sequence alignment of putative α-methylacyl-CoA racemases from *M. tuberculosis*. Protein sequences were taken from the RSCB protein databank accession numbers 2GCE (MCR), 2G04 (FAR), 5YIY (Rv3272). Active site bases (His126, Asp156, and Glu241) are highlighted in green. Sequences were aligned using Clustalw [Multiple Sequence Alignment - CLUSTALW](https://www.genome.jp/tools-bin/clustalw).

**Dose-response curves**

*
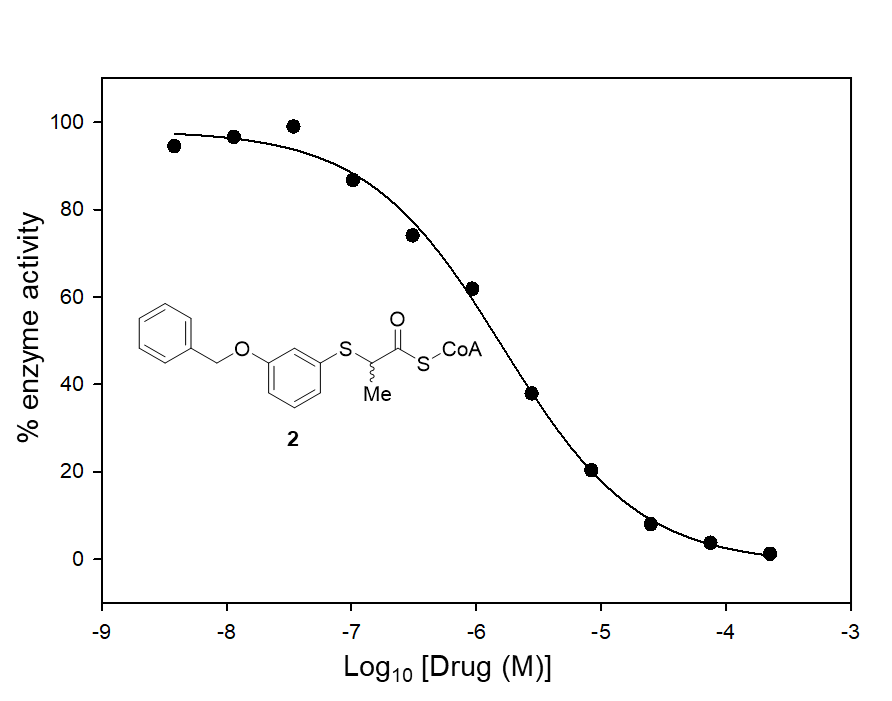
*

**Repeat 1 Coefficient Std. Error t P**

Min % -1.1030 2.7281 -0.4043 0.6981

Max % 98.1434 2.1190 46.3169 <0.0001

Log_10_ IC_50_ -5.7883 0.0575 -100.6755 <0.0001

Hillslope -0.7954 0.0817 -9.7417 <0.0001

**Repeat 2 Coefficient Std. Error t P**

Min % -1.5241 4.2467 -0.3589 0.7303

Max % 105.8137 3.0656 34.5161 <0.0001

Log_10_ IC_50_ -6.2081 0.0807 -76.9541 <0.0001

Hillslope -0.7479 0.1026 -7.2862 0.0002

**Repeat 3 Coefficient Std. Error t P**

Min % 2.7364 2.6965 1.0148 0.3440

Max % 97.7648 2.2173 44.0927 <0.0001

Log_10_ IC_50_ -6.3128 0.0605 -104.2949 <0.0001

Hillslope -0.8395 0.0945 -8.8832 <0.0001

**Figure S2: Dose-response curve for compound 2.** Data are mean % activity (2 technical repeats).

**Repeat 1 Coefficient Std. Error t P**

Min % -0.9363 1.9608 -0.4775 0.6475

Max % 107.3850 2.9942 35.8641 <0.0001

Log_10_ IC_50_ -6.3971 0.0548 -116.8099 <0.0001

Hillslope -0.6534 0.0550 -11.8711 <0.0001

**Repeat 2 Coefficient Std. Error t P**

Min % -4.2339 3.6819 -1.1499 0.2879

Max % 98.7243 5.5754 17.7073 <0.0001

Log_10_ IC_50_ -6.3716 0.1073 -59.3630 <0.0001

Hillslope -0.5594 0.0846 -6.6105 0.0003

**Repeat 3 Coefficient Std. Error t P**

Min % 0.1567 0.7520 0.2084 0.8408

Max % 100.9956 1.4891 67.8237 <0.0001

Log_10_ IC_50_ -6.6422 0.0267 -248.5279 <0.0001

Hillslope -0.7275 0.0304 -23.9457 <0.0001

**Figure S3: Dose-response curve for compound 3.** Data are mean % activity (2 technical repeats).

*
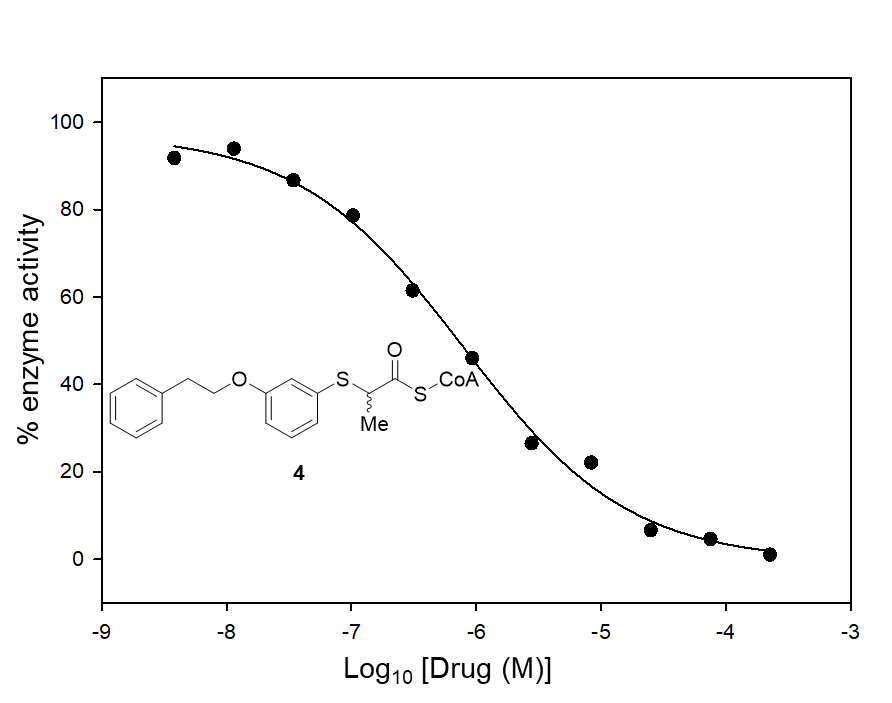
*

**Repeat 1 Coefficient Std. Error t P**

Min % -0.4789 3.0177 -0.1587 0.8784

Max % 97.3404 3.2643 29.8200 <0.0001

Log_10_ IC_50_ -6.1016 0.0757 -80.6513 <0.0001

Hillslope -0.6564 0.0798 -8.2224 <0.0001

**Repeat 2 Coefficient Std. Error t P**

Min % 0.6735 1.5899 0.4236 0.6846

Max % 97.1657 1.6167 60.0997 <0.0001

Log_10_ IC_50_ -6.0503 0.0390 -155.1708 <0.0001

Hillslope -0.8291 0.0599 -13.8355 <0.0001

**Repeat 3 Coefficient Std. Error t P**

Min % 0.8319 1.6304 0.5102 0.6256

Max % 113.9316 1.7000 67.0183 <0.0001

Log_10_ IC_50_ -6.0762 0.0345 -176.3577 <0.0001

Hillslope -0.8501 0.0552 -15.3944 <0.0001

**Figure S4: Dose-response curve for compound 4.** Data are mean % activity (2 technical repeats).

*
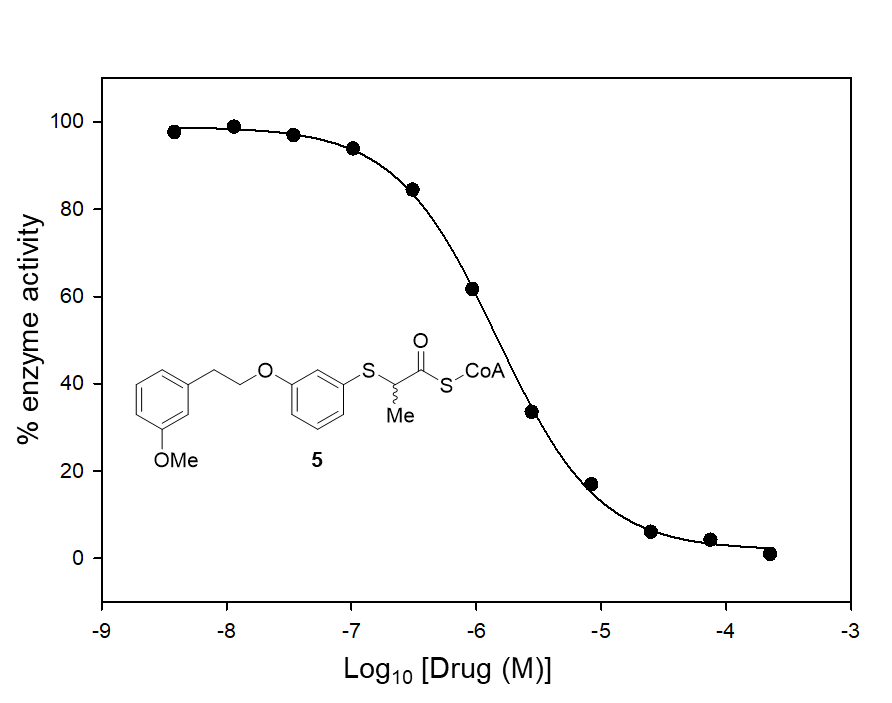
*

**Repeat 1 Coefficient Std. Error t P**

Min % 1.9097 0.8607 2.2187 0.0620

Max % 98.8979 0.7294 135.5816 <0.0001

Log_10_ IC_50_ -5.8314 0.0183 -318.4633 <0.0001

Hillslope -1.0666 0.0437 -24.4251 <0.0001

**Repeat 2 Coefficient Std. Error t P**

Min % 1.3438 0.9730 1.3811 0.2097

Max % 96.6473 0.8702 111.0577 <0.0001

Log_10_ IC_50_ -5.8997 0.0218 -270.9218 <0.0001

Hillslope -1.0398 0.0497 -20.9408 <0.0001

**Repeat 3 Coefficient Std. Error t P**

Min % -1.7690 2.9523 -0.5992 0.5679

Max % 105.7782 2.4073 43.9401 <0.0001

Log_10_ IC_50_ -5.8272 0.0583 -99.9041 <0.0001

Hillslope -0.8398 0.0911 -9.2203 <0.0001

**Figure S5: Dose-response curve for compound 5.** Data are mean % activity (2 technical repeats).

*
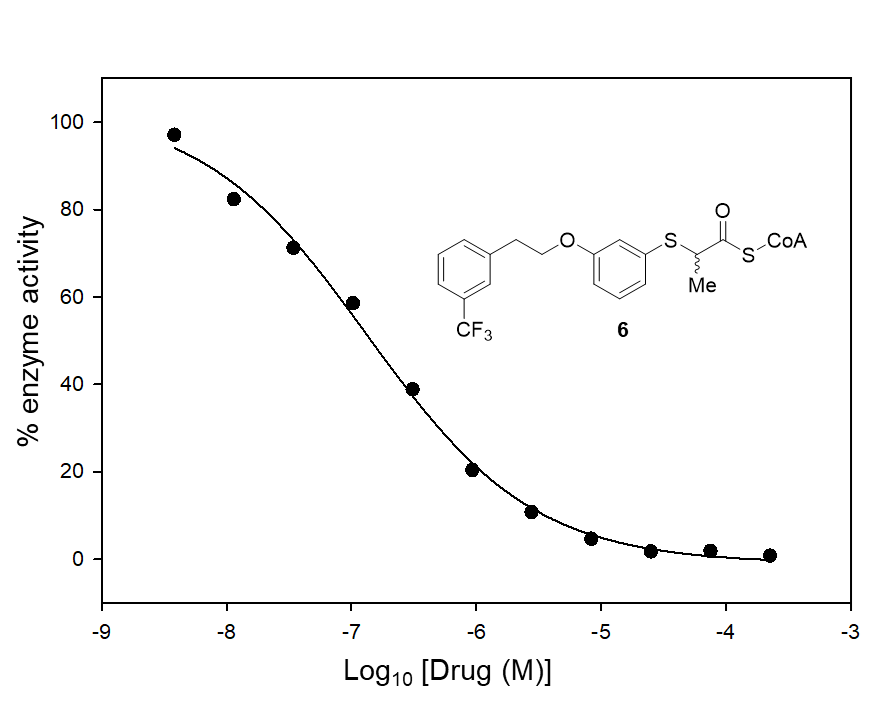
*

**Repeat 1 Coefficient Std. Error t P**

Min % -0.9803 1.8405 -0.5326 0.6108

Max % 103.3829 5.0807 20.3480 <0.0001

Log_10_ IC_50_ -6.8729 0.0843 -81.5274 <0.0001

Hillslope -0.6535 0.0709 -9.2127 <0.0001

**Repeat 2 Coefficient Std. Error t P**

Min % 1.2304 1.2003 1.0251 0.3394

Max % 96.3798 3.6372 26.4985 <0.0001

Log_10_ IC_50_ -6.9630 0.0637 -109.2942 <0.0001

Hillslope -0.6960 0.0584 -11.9207 <0.0001

**Repeat 3 Coefficient Std. Error t P**

Min % 2.6614 1.7355 1.5335 0.1690

Max % 100.3852 2.5824 38.8733 <0.0001

Log_10_ IC_50_ -6.5001 0.0489 -132.8607 <0.0001

Hillslope -1.0454 0.1115 -9.3747 <0.0001

**Figure S6: Dose-response curve for compound 6.** Data are mean % activity (2 technical repeats).


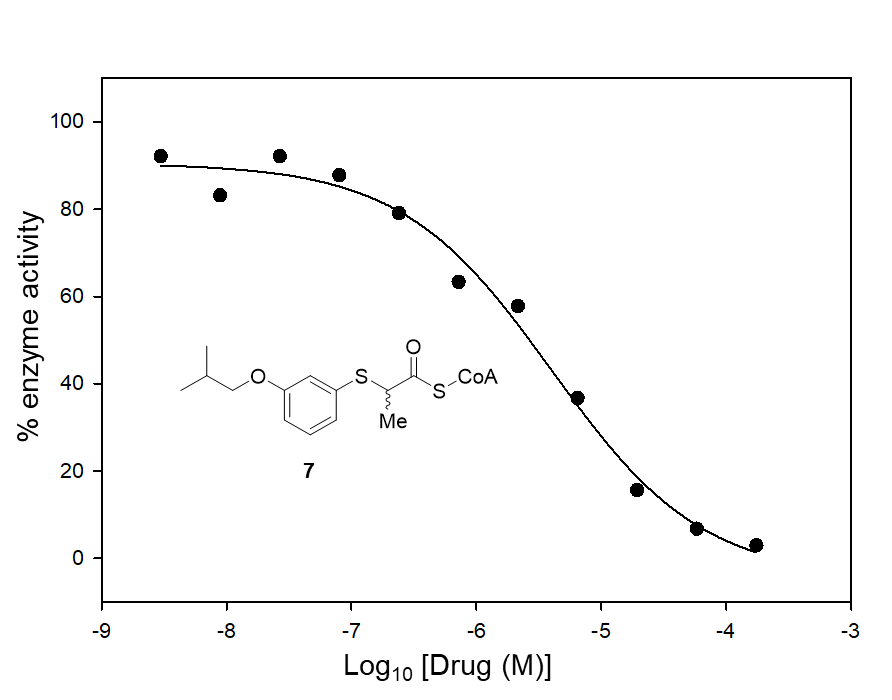


**Repeat 1 Coefficient Std. Error t P**

Min % -4.5229 7.1082 -0.6363 0.5448

Max % 90.5377 3.0157 30.0226 <0.0001

Log_10_ IC_50_ -5.3942 0.1296 -41.6234 <0.0001

Hillslope -0.7207 0.1381 -5.2198 0.0012

**Repeat 2 Coefficient Std. Error t P**

Min % -6.3244 3.3994 -1.8605 0.1051

Max % 102.5775 1.5573 65.8678 <0.0001

Log_10_ IC_50_ -5.4794 0.0561 -97.6518 <0.0001

Hillslope -0.6683 0.0534 -12.5183 <0.0001

**Repeat 3 Coefficient Std. Error t P**

Min % -4.9323 4.7519 -1.0380 0.3338

Max % 94.2962 1.8858 50.0021 <0.0001

Log_10_ IC_50_ -5.3226 0.0804 -66.177 <0.0001

Hillslope -0.7565 0.0919 -8.2283 <0.0001

**Figure S7: Dose-response curve for compound 7.** Data are mean % activity (2 technical repeats).

*
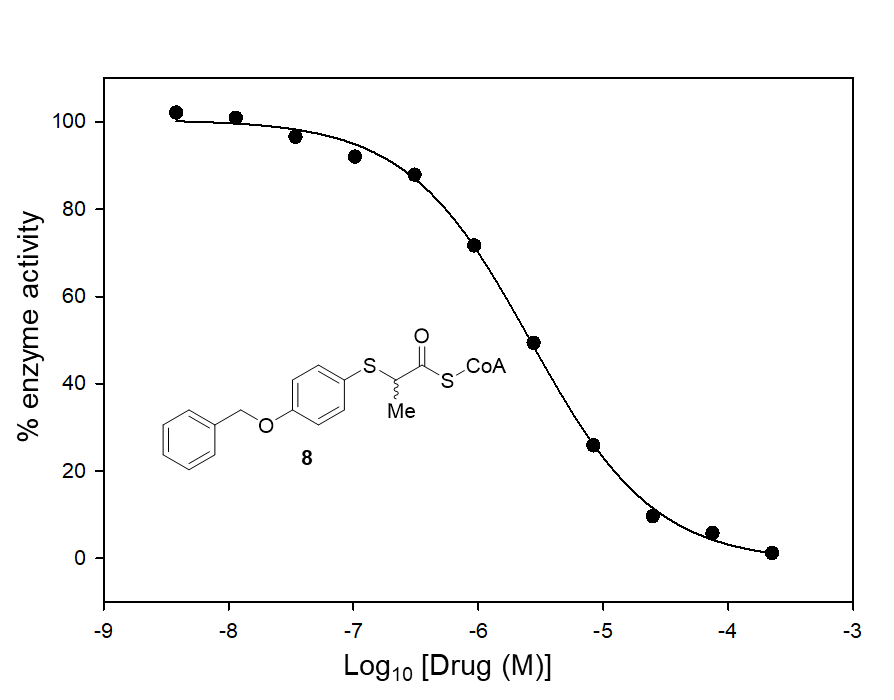
*

**Repeat 1 Coefficient Std. Error t P**

Min % -0.8366 1.8304 -0.4571 0.6615

Max % 100.5015 1.1774 85.3576 <0.0001

Log_10_ IC_50_ -5.5814 0.0343 -162.6202 <0.0001

Hillslope -0.8760 0.0565 -15.4924 <0.0001

**Repeat 2 Coefficient Std. Error t P**

Min % 0.2347 1.4756 0.1591 0.8781

Max % 98.3222 0.9819 100.1392 <0.0001

Log_10_ IC_50_ -5.5830 0.0283 -197.0011 <0.0001

Hillslope -0.9668 0.0559 -17.2881 <0.0001

**Repeat 3 Coefficient Std. Error t P**

Min % 0.0581 0.4861 0.1195 0.9082

Max % 96.0781 0.3163 303.7686 <0.0001

Log_10_ IC_50_ -5.5531 0.0094 -591.1767 <0.0001

Hillslope -0.9845 0.0191 -51.4769 <0.0001

**Figure S8: Dose-response curve for compound 8.** Data are mean % activity (2 technical repeats).

*
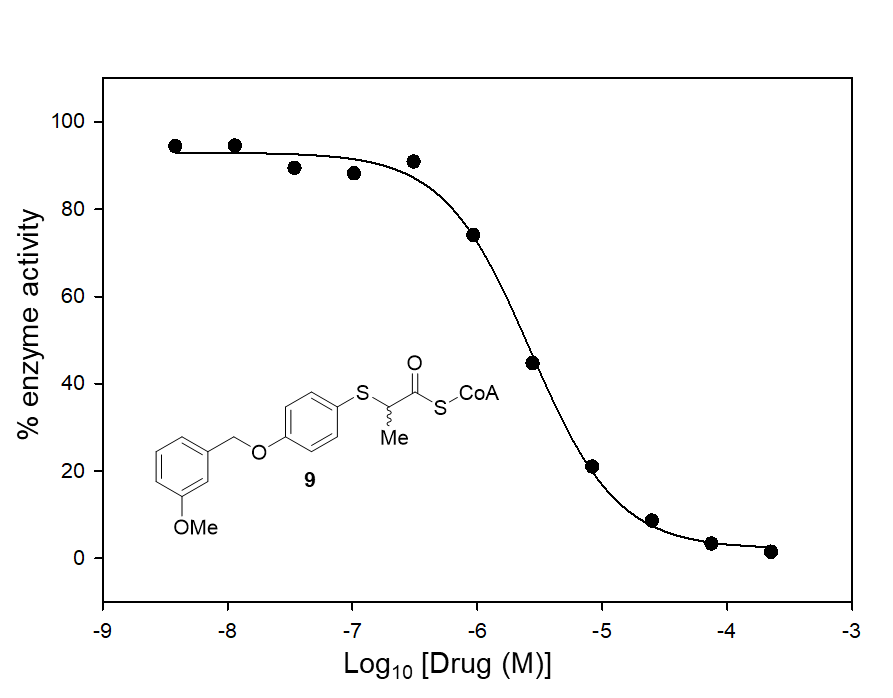
*

**Repeat 1 Coefficient Std. Error t P**

Min % 2.2166 1.8791 1.1796 0.2767

Max % 92.9975 1.3366 69.5751 <0.0001

Log_10_ IC_50_ -5.5739 0.0371 -150.0407 <0.0001

Hillslope -1.2477 0.1186 -10.5159 <0.0001

**Repeat 2 Coefficient Std. Error t P**

Min % 0.0146 2.0149 0.0073 0.9944

Max % 103.4821 1.3823 74.8612 <0.0001

Log_10_ IC_50_ -5.6485 0.0381 -148.1257 <0.0001

Hillslope -0.8652 0.0619 -13.9834 <0.0001

**Repeat 3 Coefficient Std. Error t P**

Min % 0.9734 1.5976 0.6093 0.5616

Max % 92.7721 1.0774 86.1049 <0.0001

Log_10_ IC_50_ -5.5470 0.0316 -175.5663 <0.0001

Hillslope -1.1180 0.0817 -13.6895 <0.0001

**Figure S9: Dose-response curve for compound 9.** Data are mean % activity (2 technical repeats).

*
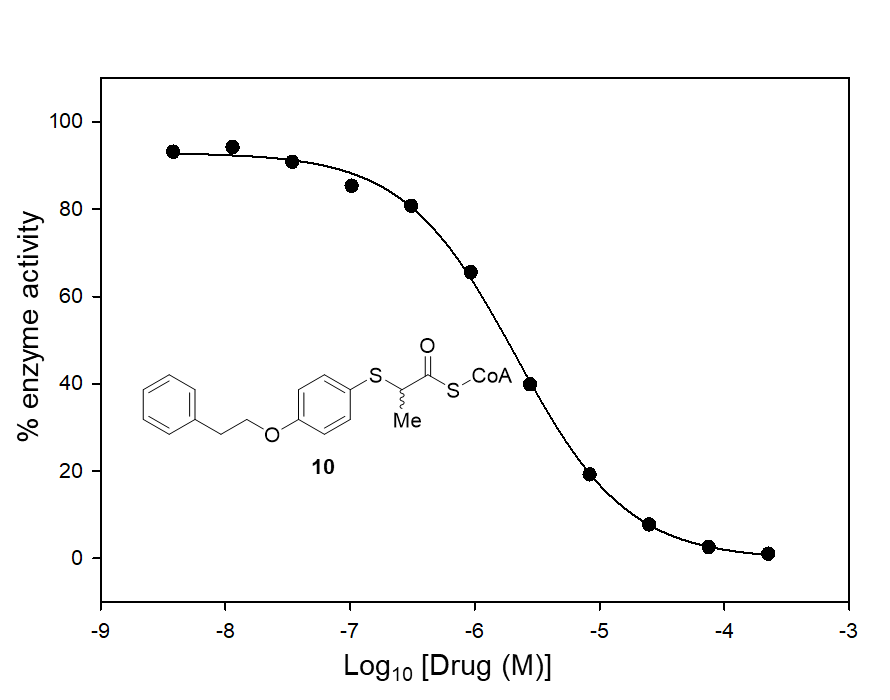
*

**Repeat 1 Coefficient Std. Error t P**

Min % -0.2465 1.2204 -0.2020 0.8457

Max % 92.9368 0.8815 105.4267 <0.0001

Log_10_ IC_50_ -5.6705 0.0256 -221.2483 <0.0001

Hillslope -0.9669 0.0509 -19.0077 <0.0001

**Repeat 2 Coefficient Std. Error t P**

Min % 3.4309 1.4853 2.3100 0.0542

Max % 90.6327 1.0335 87.6945 <0.0001

Log_10_ IC_50_ -5.5205 0.0293 -188.3252 <0.0001

Hillslope -1.3506 0.1093 -12.3612 <0.0001

**Repeat 3 Coefficient Std. Error t P**

Min % 1.9814 1.1176 1.7729 0.1195

Max % 97.8177 0.8000 122.2696 <0.0001

Log_10_ IC_50_ -5.6037 0.0215 -260.4647 <0.0001

Hillslope -1.1651 0.0603 -19.3359 <0.0001

**Figure S10: Dose-response curve for compound 10.** Data are mean % activity (2 technical repeats).


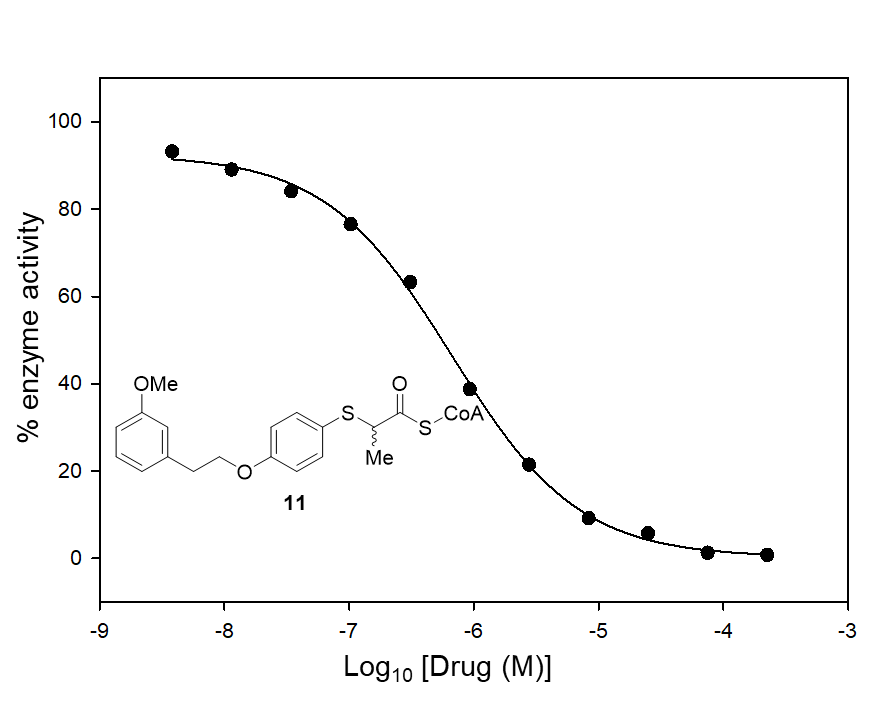


**Repeat 1 Coefficient Std. Error t P**

Min % 0.2507 1.1069 0.2265 0.8273

Max % 92.5335 1.2701 72.8563 <0.0001

Log_10_ IC_50_ -6.1746 0.0301 -205.1559 <0.0001

Hillslope -0.8553 0.0486 -17.5861 <0.0001

**Repeat 2 Coefficient Std. Error t P**

Min % 1.0359 2.3289 0.4448 0.6699

Max % 88.3414 2.0633 42.8149 <0.0001

Log_10_ IC_50_ -5.8812 0.0557 -105.5601 <0.0001

Hillslope -1.1060 0.1421 -7.7813 0.0001

**Repeat 3 Coefficient Std. Error t P**

Min % -1.9106 2.8199 -0.6775 0.5198

Max % 111.5877 3.4868 32.0026 <0.0001

Log_10_ IC_50_ -6.2231 0.0658 -94.5807 <0.0001

Hillslope -0.6966 0.0755 -9.2261 <0.0001

**Figure S11: Dose-response curve for compound 11.** Data are mean % activity (2 technical repeats).


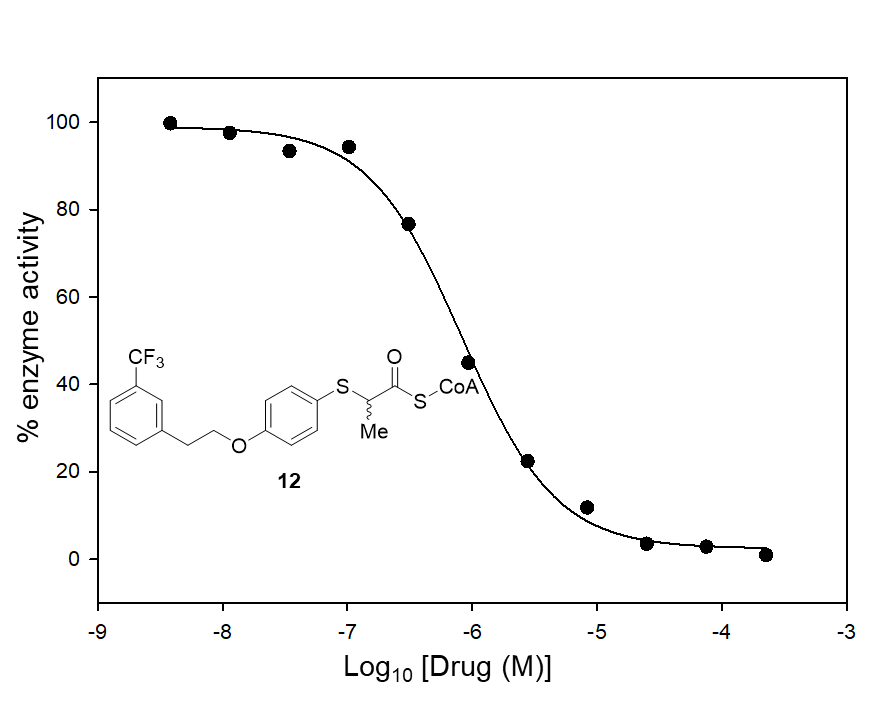


**Repeat 1 Coefficient Std. Error t P**

Min % 2.4547 1.5456 1.5882 0.1563

Max % 98.9383 1.6043 61.6701 <0.0001

Log_10_ IC_50_ -6.0819 0.0357 -170.2189 <0.0001

Hillslope -1.1570 0.0993 -11.6522 <0.0001

**Repeat 2 Coefficient Std. Error t P**

Min % 0.7925 1.2110 0.6544 0.5338

Max % 96.1278 1.3401 71.7332 <0.0001

Log_10_ IC_50_ -6.1514 0.0304 -202.3936 <0.0001

Hillslope -1.0035 0.0650 -15.4346 <0.0001

**Repeat 3 Coefficient Std. Error t P**

Min % 2.1946 2.3233 0.9446 0.3763

Max % 100.8644 2.2398 45.0318 <0.0001

Log_10_ IC_50_ -5.9814 0.0480 -124.5896 <0.0001

Hillslope -1.3522 0.1799 -7.5187 0.0001

**Figure S12: Dose-response curve for compound 12.** Data are mean % activity (2 technical repeats).


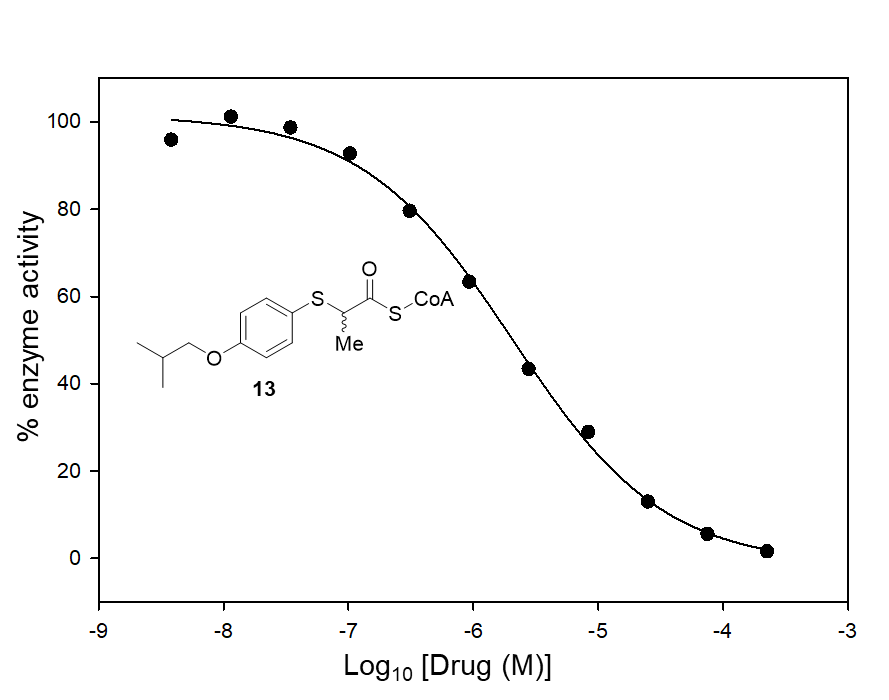


**Repeat 1 Coefficient Std. Error t P**

Min % -1.4547 3.0001 -0.4849 0.6426

Max % 101.5547 2.0358 49.8834 <0.0001

Log_10_ IC_50_ -5.6834 0.0582 -97.7229 <0.0001

Hillslope -0.7177 0.0686 -10.4692 <0.0001

**Repeat 2 Coefficient Std. Error t P**

Min % 1.6442 2.2001 0.7473 0.4792

Max % 99.3712 1.5919 62.4210 <0.0001

Log_10_ IC_50_ -5.7139 0.0455 -125.5996 <0.0001

Hillslope -0.8209 0.0677 -12.1191 <0.0001

**Repeat 3 Coefficient Std. Error t P**

Min % -0.7140 1.2921 -0.5526 0.5977

Max % 97.9962 0.9874 99.2450 <0.0001

Log_10_ IC_50_ -5.7862 0.0274 -211.5601 <0.0001

Hillslope -0.7335 0.0339 -21.6271 <0.0001

**Figure S13: Dose-response curve for compound 13.** Data are mean % activity (2 technical repeats).


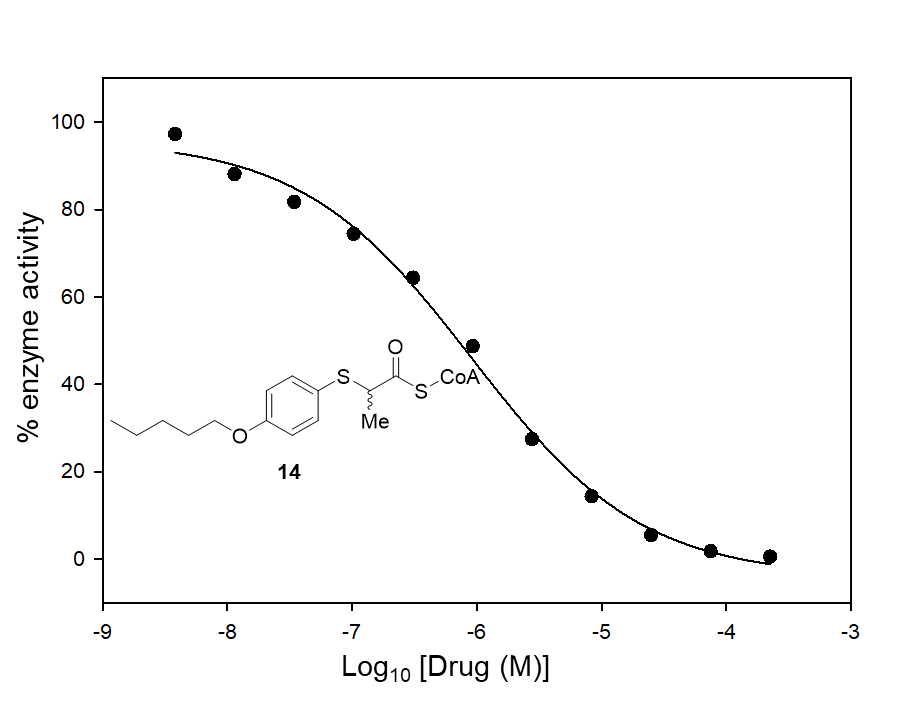


**Repeat 1 Coefficient Std. Error t P**

Min % -4.0855 3.1796 -1.2849 0.2397

Max % 96.0022 3.2022 29.9804 <0.0001

Log_10_ IC_50_ -6.0395 0.0749 -80.6638 <0.0001

Hillslope -0.6352 0.0753 -8.4378 <0.0001

**Repeat 2 Coefficient Std. Error t P**

Min % 1.2871 2.6434 0.4869 0.6412

Max % 98.2019 2.9863 32.8839 <0.0001

Log_10_ IC_50_ -6.1613 0.0676 -91.0922 <0.0001

Hillslope -0.8783 0.1144 -7.6754 0.0001

**Repeat 3 Coefficient Std. Error t P**

Min % -0.2422 1.2992 -0.1864 0.8574

Max % 102.2888 1.3532 75.5914 <0.0001

Log_10_ IC_50_ -6.0728 0.0305 -199.2479 <0.0001

Hillslope -0.7880 0.0430 -18.3112 <0.0001

**Figure S14: Dose-response curve for compound 14.** Data are mean % activity (2 technical repeats).

**Jump dilution experiments to test reversibility of inhibition**


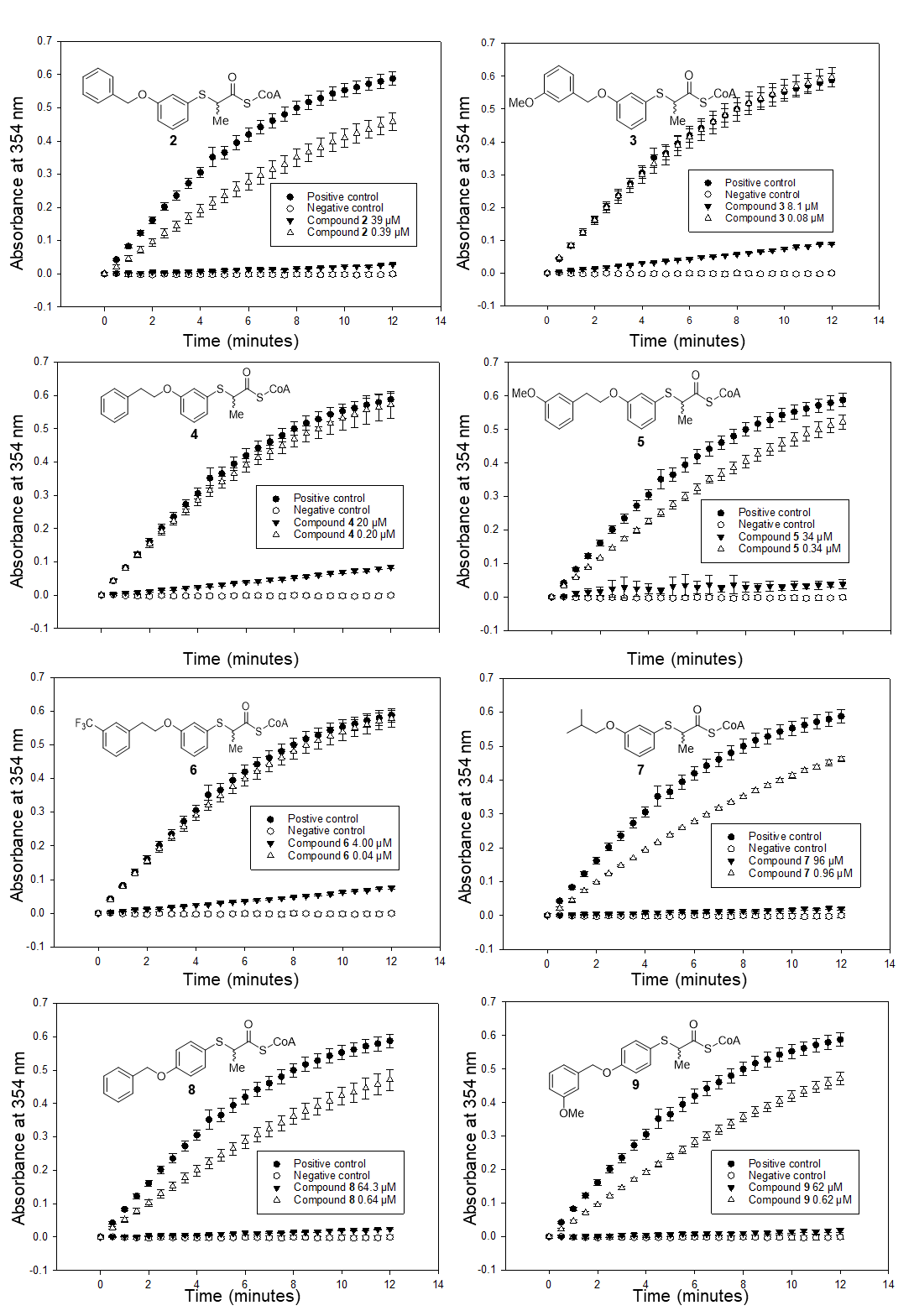


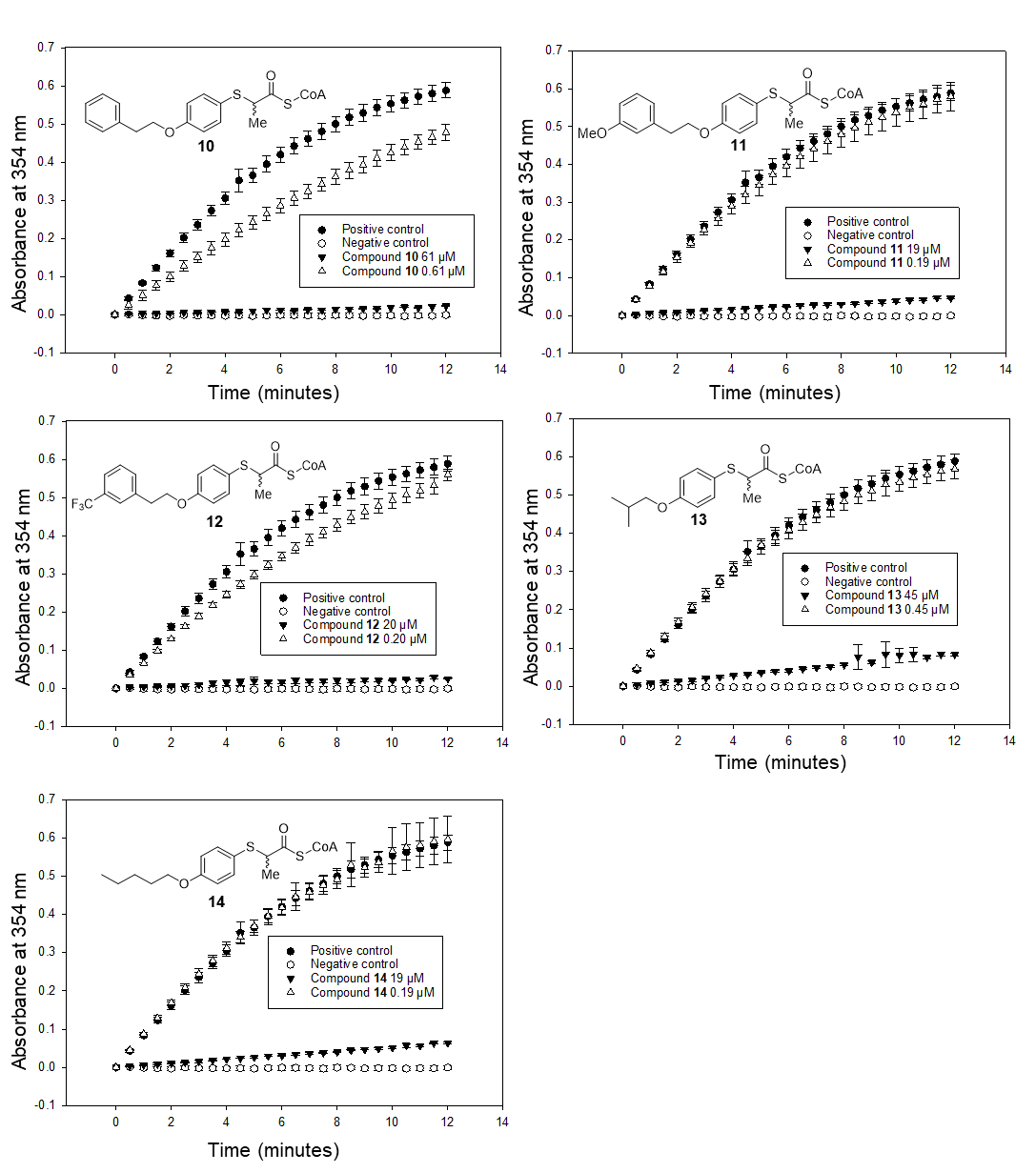


**Figure S15:** Jump dilution experiments to determine whether inhibition by compounds **2** to **14** are reversible. Data are means ± SD for three technical repeats. These experiments show that all compounds are reversible inhibitors.

**Determination of *K*_i_ value for 6**

**
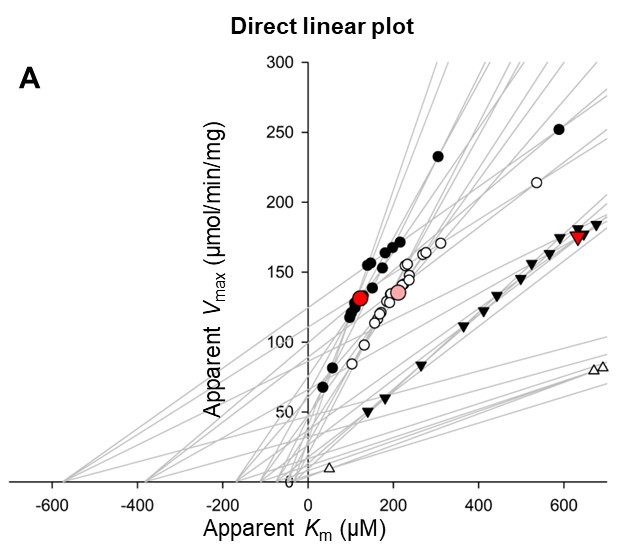
**

**
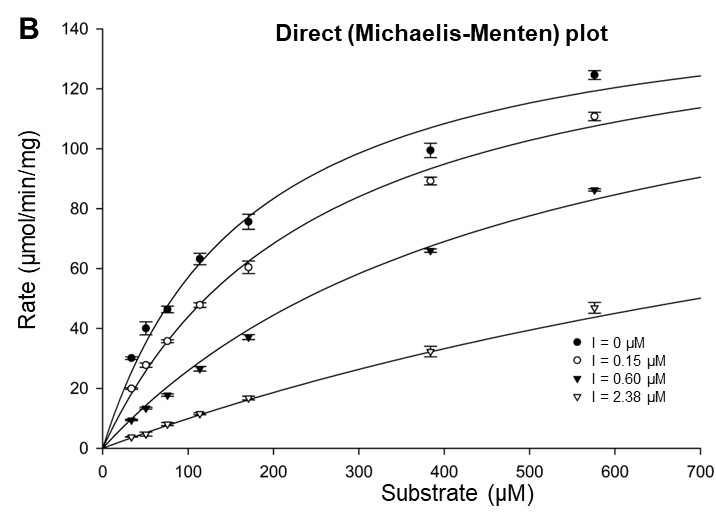
**

**
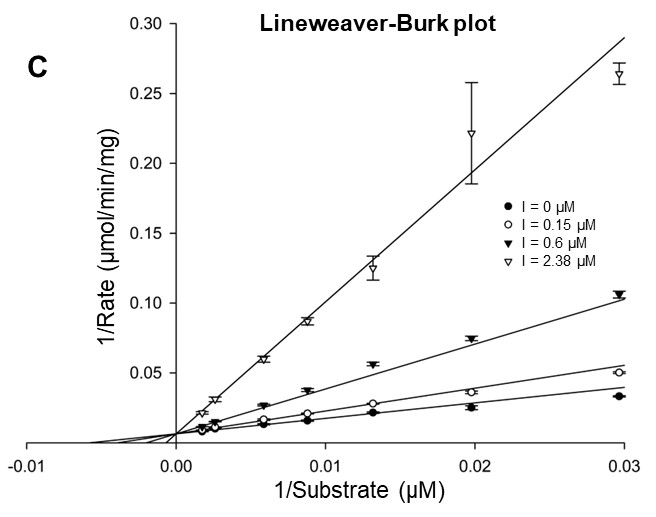
**

**
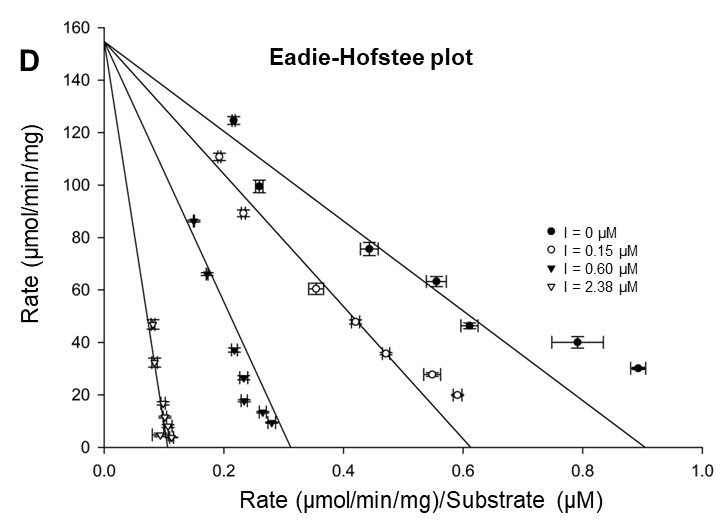
**

**
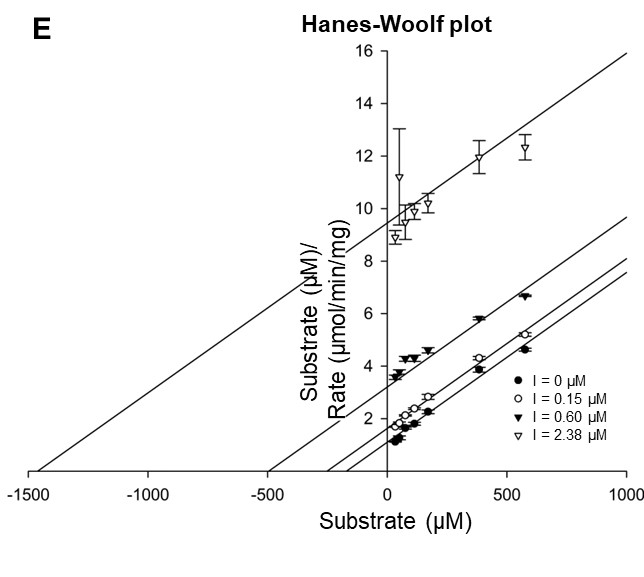
**

**
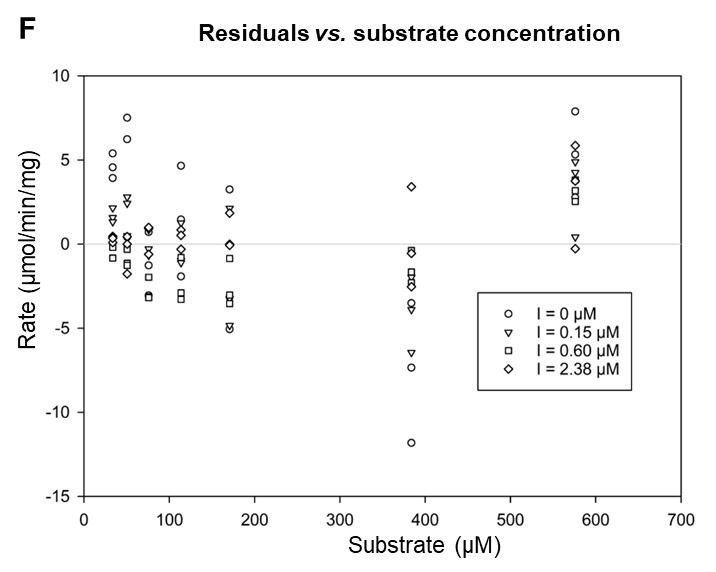
**

**
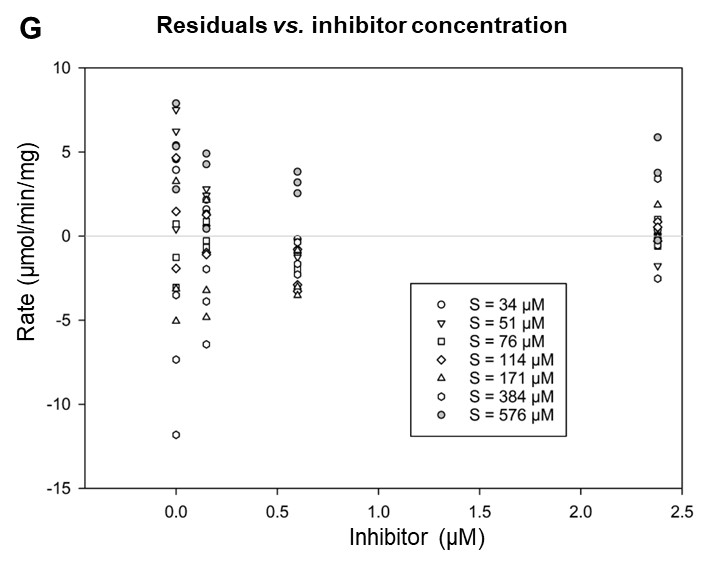
**

**Parameters (repeat 1)**

Value ± Std. Error 95% Conf. Interval

*V*_max_ (μmol/min/mg) 147.2543 2.5453 142.1899 to 152.3187

*K*_m_ (μM) 118.5720 5.7206 107.1896 to 129.9545

*K*_i_ (μM) 0.1900 9.468e-3 0.1712 to 0.2089

**Goodness of Fit**

Degrees of Freedom 81

AICc 226.154

R² 0.989

Sum of Squares 1,120.869

Sy.x 3.720

Runs Test p Value < .001

**Data**

Number of x values 28

Number of replicates 3

Total number of values 84

Number of missing values 0

**Parameters (repeat 2)**

Value ± Std. Error 95% Conf. Interval

*V*_max_ (μmol/min/mg) 154.7148 2.8276 149.0886 to 160.3410

*K*_m_ (μM) 171.0430 7.7476 155.6274 to 186.4586

*K*_i_ (μM) 0.3153 1.485e-2 0.2858 to 0.3449

Goodness of Fit

Degrees of Freedom 81

AICc 208.203

R² 0.990

Sum of Squares 905.201

Sy.x 3.343

Runs Test p Value 0.500

Data

Number of x values 28

Number of replicates 3

Total number of values 84

Number of missing values 0

**Parameters (repeat 3)**

Value ± Std. Error 95% Conf. Interval

*V*_max_ (μmol/min/mg) 144.4306 2.8431 138.7737 to 150.0876

*K*_m_ (μM) 119.6380 6.5645 106.5764 to 132.6997

*K*_i_ (μM) 0.2098 1.203e-2 0.1858 to 0.2337

**Goodness of Fit**

Degrees of Freedom 81

AICc -494.043

R² 0.985

Sum of Squares 0.212

Sy.x 5.114e-2

Runs Test p Value < 0.001

**Data**

Number of x values 28

Number of replicates 3

Total number of values 84

Number of missing values 0

**Figure S16:** Determination of inhibition mode for **6**. Rates were determined using ICEKAT (42,43) and data was analysed using SigmaPlot 15.0 (45). A. to G. different kinetic plots used to analyse the data for a representative independent repeat. Data in B to E are means ± SD for three technical repeats for each inhibitor and substrate concentration.
